# Microglial phagolysosome dysfunction and altered neural communication amplify phenotypic severity in Prader-Willi Syndrome with larger deletion

**DOI:** 10.1007/s00401-024-02714-0

**Published:** 2024-03-31

**Authors:** Felipe Correa-da-Silva, Jenny Carter, Xin-Yuan Wang, Rui Sun, Ekta Pathak, José Manuel Monroy Kuhn, Sonja C. Schriever, Clarissa M. Maya-Monteiro, Han Jiao, Martin J. Kalsbeek, Pedro M. M. Moraes-Vieira, Johan J. P. Gille, Margje Sinnema, Constance T. R. M. Stumpel, Leopold M. G. Curfs, Dirk Jan Stenvers, Paul T. Pfluger, Dominik Lutter, Alberto M. Pereira, Andries Kalsbeek, Eric Fliers, Dick F. Swaab, Lawrence Wilkinson, Yuanqing Gao, Chun-Xia Yi

**Affiliations:** 1 Department of Endocrinology and Metabolism, Amsterdam University Medical Centers, Location AMC. University of Amsterdam, Meibergdreef 9, 1105AZ Amsterdam, The Netherlands; 2 Amsterdam Gastroenterology Endocrinology and Metabolism, Amsterdam, The Netherlands; 3 Endocrine Laboratory, Department of Laboratory Medicine, Amsterdam University Medical Centers, Location AMC, Amsterdam, The Netherlands; 4 Netherlands Institute for Neuroscience, Amsterdam, The Netherlands; 5 Neuroscience and Mental Health Innovation Institute, MRC Centre for Neuropsychiatric Genetic and Genomics, School of Medicine, Cardiff University, Cardiff, UK; 6 Key Laboratory of Cardiovascular and Cerebrovascular Medicine, School of Pharmacy, Nanjing Medical University, Nanjing, China; 7 Computational Discovery Unit, Institute for Diabetes and Obesity, Helmholtz Zentrum München, Neuherberg, Germany; 8 German Center for Diabetes Research (DZD), Neuherberg, Germany; 9 Research Unit NeuroBiology of Diabetes, Institute for Diabetes and Obesity, Helmholtz Zentrum München, Neuherberg, Germany; 10 Laboratory of Immunopharmacology, Oswaldo Cruz Institute, FIOCRUZ, Rio de Janeiro, Brazil; 11 Laboratory of Immunometabolism, Department of Genetics, Evolution, Microbiology and Immunology, Institute of Biology, University of Campinas, Campinas, São Paulo Brazil; 12 Department of Clinical Genetics, Amsterdam University Medical Centers, location VUMC. University of Amsterdam, Amsterdam, The Netherlands; 13 Department of Clinical Genetics, Maastricht University Medical Center, Maastricht, The Netherlands; 14 Governor Kremers Centre, Maastricht University Medical Centre, Maastricht, The Netherlands; 15 Division of Neurobiology of Diabetes, TUM School of Medicine, Technical University of Munich, Munich, Germany

**Keywords:** Hypothalamus, Fornix, Microglia, Immunosurveillance, Myelin, Glymphatic system, Oxytocin

## Abstract

Prader-Willi Syndrome (PWS) is a rare neurodevelopmental disorder of genetic etiology, characterized by paternal deletion of genes located at chromosome 15 in 70% of cases. Two distinct genetic subtypes of PWS deletions are characterized, where type I (PWS T1) carries four extra haploinsufficient genes compared to type II (PWS T2). PWS T1 individuals display more pronounced physiological and cognitive abnormalities than PWS T2, yet the exact neuropathological mechanisms behind these differences remain unclear. Our study employed postmortem hypothalamic tissues from PWS T1 and T2 individuals, conducting transcriptomic analyses and cell-specific protein profiling in white matter, neurons, and glial cells to unravel the cellular and molecular basis of phenotypic severity in PWS sub-genotypes. In PWS T1, key pathways for cell structure, integrity, and neuronal communication are notably diminished, while glymphatic system activity is heightened compared to PWS T2. The microglial defect in PWS T1 appears to stem from gene haploinsufficiency, as global and myeloid-specific *Cyfip1* haploinsufficiency in murine models demonstrated. Our findings emphasize microglial phagolysosome dysfunction and altered neural communication as crucial contributors to the severity of PWS T1’s phenotype.

**Supplementary Information:**

The online version contains supplementary material available at 10.1007/s00401-024-02714-0.

## Introduction

Prader-Willi Syndrome (PWS) is a neurodevelopmental genetic disorder caused by the lack of gene expression in chromosome 15q11.2-q13 region [7]. Roughly 70% of cases are attributed to the deletion of a segment on the paternal chromosome, leading to two primary sub-genotypes: Type I (PWS T1) and Type II (PWS T2) [52]. PWS T1 deletion involves the haploinsufficiency of four additional protein-encoding genes, distinguishing it from PWS T2 [6]. The clinical spectrum of PWS encompasses a broad array of features, including morbid obesity, endocrine deficiencies, intellectual disability, hindered linguistic and motor milestones, primarily arising from hypothalamic dysfunction [6, 11, 20, 35]. In addition, more extensive genetic deletion (PWS T1) leads to heightened clinical manifestations such as compulsive behaviors, obsessive thoughts, self-injury, cognitive impairment, and disruptions in visual processing [6, 11, 20, 35]. Yet, the precise neuropathological mechanisms tying genotypic differences to phenotypic expression remain elusive. A better understanding of these mechanisms holds promise to develop tailored therapy for PWS patients with distinct sub-genotypes.

The hypothalamus emerges as a pivotal brain region governing PWS pathophysiology, as evident in recent human observations and animal models [11, 39]. Findings from hypothalamic tissue specimens from PWS subjects pinpoint the specific loss of neuronal populations residing within the hypothalamus, tightly associated with metabolic and behavioral deviations [11]. Neuroimaging studies accentuate compromised hypothalamic neural networks in PWS patients, especially in regions linked to feeding behavior [21]. In addition, disruption of white matter connecting hypothalamus and cortex appears correlated with impaired satiety in PWS patients [4]. Nevertheless, whether these hypothalamic dysfunctions diverge between PWS T1 and T2 sub-genotypes remains uncertain.

In this study, we dissected the genotype-based cellular and molecular distinctions between PWS T1 and T2. Leveraging postmortem brain tissues of PWS patients, comprising three with a T1 deletion and seven with a T2 deletion, we conducted comparisons with control subjects without PWS deletion. Our transcriptomic analyses of hypothalamic tissues unveil significant downregulation of genes implicated in controlling cell structure, integrity, morphology, and neuronal communication in PWS T1. Consistent with these alterations, we observed pronounced microglial dysmorphism and impaired phagolysosome activity in the brains of PWS T1, coupled with heightened expression of the glymphatic component aquaporin 4 (AQP4). Employing rat and mouse models, we validated that microglial dysmorphism stems from the dysfunctional cytoplasmic Fragile X Messenger Ribonucleoprotein 1 (*FMR1*) interacting protein 1 (*CYFIP1*), one of the four additional haploinsufficient genes exclusive to PWS T1 [8]. Moreover, we detected compromised myelin integrity in the fornix and decreased synaptophysin expression in the hypothalamus. Collectively, our findings suggest that microglial dysfunction and altered neural communication emerge as principal factors potentially amplifying phenotypic severity in patients with PWS T1 deletion.

## Materials and methods

### Subject information

Postmortem hypothalamic tissues were collected from 10 individuals diagnosed with PWS and 32 matched controls, through collaboration with the Netherlands Brain Bank (NBB). Controls were defined as subjects without PWS genomic deletion and without known endocrine or metabolic pathologies. Exclusion criteria included individuals who died from brain tumours, encephalitis, or exhibited mild to severe dementia (Braak stages 3–6) [2]. In addition, individuals with known neurological or psychiatric conditions and those who had received anti-inflammatory medication within three months prior to demise, due to recognized confounding factors, were also excluded. Subjects whose Braak stage analysis was unavailable were incorporated as controls, provided their medical records did not indicate severe dementia. Genetic subtyping of PWS T1 and T2 was executed via employment of the Multiplex Ligation-dependent Probe Amplification (MLPA) assay. Genetic subtypes of PWS encompassing maternal uniparental disomy and atypical deletion with imprinting defect [37] were not encompassed within the scope of this study. All groups, including sub-genotypes within the PWS group, were matched for sex, age, fixation time, and postmortem delay (Supplementary Table 1). A comprehensive overview of clinicopathological information for all subjects is provided in Table 1.

**Table 1:** Subjects group characteristics

	Control	PWS T1	PWS T2
Age (years)	33.8 ± 25.8	17.8 ± 27.1	34.3 ± 20.0
Female (N, %)	16 (50)	1 (33.3)	3 (42.8)
Male (N, %)	16 (50)	2 (66.7)	4 (57.2)
Postmortem delay (hours)	25.3 ± 22.2	9.8	23.4 ± 20.1
Body mass index (kg/m^2^)	24.0 ± 6.5	28 ± 15.5	39.2 ± 21.5 *

N, total number of individuals. Controls (n = 32), PWS T1 (n = 3), and PWS T2 (n = 7). Data are represented as the mean ± SD. * p < 0.05 v.s. control, obtained by Kruskal–Wallis test followed by Dunn’s test

### Animal models

Animal experiments were conducted under controlled conditions with a 12-h light/dark cycle, stable temperature, standard chow diet, and ad libitum access to water and food. Global *Cyfip1* heterozygous (*Cyfip1* +/−) rats were generated and genotyped as previously described [44]. Adult male rats between 8 and 12 weeks of age were used in the experiments. An inducible myeloid cells-specific *Cyfip1* heterozygous knockdown mouse line was generated by crossbreeding *Cyfip1*^*fl*+*/*+^ mice with *Cx3cr1*^*Cre−ERT*^ mice and administering tamoxifen to induce haploinsufficiency of the *Cyfip1* gene (*Cx3cr1*^*Cre−ERT*+/−^ *Cyfip1*^*fl*+/−^), using *Cx3cr1*^*Cre−ERT*+/−^ *Cyfip1*^*fl−/−*^ mice as controls. Tamoxifen was administered for 3 days during postnatal days 5–7 (2 mg/ml, 50 µl/day). Genotyping confirmed the *Cyfip1* delta band after *Cre*-mediated recombination. Male mice at 32 weeks of age were used for the experiments.

### Immunohistochemistry and immunofluorescence

For studies with human brains, after autopsy, the dissected hypothalami were immersed in 10% phosphate-buffered formalin at room temperature. Brain tissue was ethanol-dehydrated, toluene-cleared, and paraffin-embedded. All the formalin-fixed paraffin-embedded (FFPE) hypothalamic tissue was coronally serially sectioned from rostral to caudal at 6 μm. Immunohistochemical or immunofluorescent staining of ionized calcium-binding adapter molecule 1 (Iba1), Transmembrane Protein 119 (TMEM 119), P2Y12 receptor (P2Y12R), proteolipid protein (PLP), synaptophysin, proopiomelanocortin (POMC), Neuropeptide Y (NPY), arginine-vasopressin (AVP), oxytocin (OXT), cluster of differentiation 68 (CD68), cathepsin S (CTSS), Lysosomal-associated membrane protein 1(LAMP1), aquaporin 4 (AQP4), and alpha-smooth muscle actin (alpha-SMA) were detected using specific antibodies (Supplementary Table 2) in the human brain. Immunohistochemical staining of Iba1 was also analysed in the rodent brains. Immunofluorescence for colocalization of CD68/Iba1, CTSS/Iba1, LAMP1/Iba1, PLP/Iba1 or AQP4/alpha-SMA were performed in human brain or mouse brain sections. DAPI nuclei-counterstaining was selectively performed in both the cases (Supplementary materials and methods).

### RNA isolation, sequencing and data analysis

RNA was isolated from FFPE human hypothalamic tissues following a detailed procedure (Supplementary materials and methods). RNA concentration was measured using Bioanalyzer RNA Pico Chips, and cDNA libraries were constructed for next-generation RNAseq analysis on an Illumina sequence platform (Novogene, Cambridge, UK). The quality control of the sequencing data was performed accordingly (Supplementary Table 3). The RNA transcript expression was quantified with Kallisto (default settings, v0.46.1) [3] using the Ensembl human GRCh38 reference transcriptome. The gene-level count matrix was created using Tximport [46] and DESeq2 was used for differential gene expression analysis [30]. After estimation and DESeq2 normalization of raw read counts, we performed PCA analysis to test for potential bias in the data by batch (of RNA isolation), sex, subgenotype groups, age and postmortem delay. For none of these co-factors a conspicuous source of bias could be identified (Supplementary Fig. 1 and 2). Differentially expressed genes (DEGs) were obtained between conditions (Control vs. PWS T1, Control vs. PWS T2 and PWS T1 vs. PWS T2), after performing false discovery rate (FDR) correction using (Benjamini-Hochberg) an adjusted p < 0.05 was used to identify significantly differential expressed genes (deposited data). A REACTOME enrichment pathway analysis was performed with the R package ReactomePA [56].

### Statistical analysis

All data are presented as the mean ± SEM. D’Agostino and Pearson normality tests were performed to determine the data normality, and appropriate statistical tests were selected based on data distribution. For human parameters, Kruskal–Wallis test followed by Dunn’s test was used for non-normally distributed data, while Student’s t-test was used for normally distributed data in rodent samples. Multiple testing was controlled using the Benjamini–Hochberg criterion. Statistical significance was set at 0.05, and all analyses were conducted using GraphPad Prism 8.12.

### Study approval

For human subjects, consent was obtained from donors or their legal guardians for brain autopsy and use of medical records and brain tissue for research purposes [27]. For animal experiments, all studies performed with *Cyfip1* heterozygous rats were in accordance with institutional animal welfare, ethical, and ARRIVE guidelines, and under the UK Home Office License PPL 30/3135 (Animals (Scientific Procedures) Act 1986). All studies performed with microglial *Cyfip1* knockout mice were in accordance with the ethical policies and procedures approved by the Animal Core Facility of Nanjing Medical University (IACUC-2101036).

## Results

### PWS T1 deletion is associated with impaired cellular integrity in the hypothalamus

**Fig. 1:**
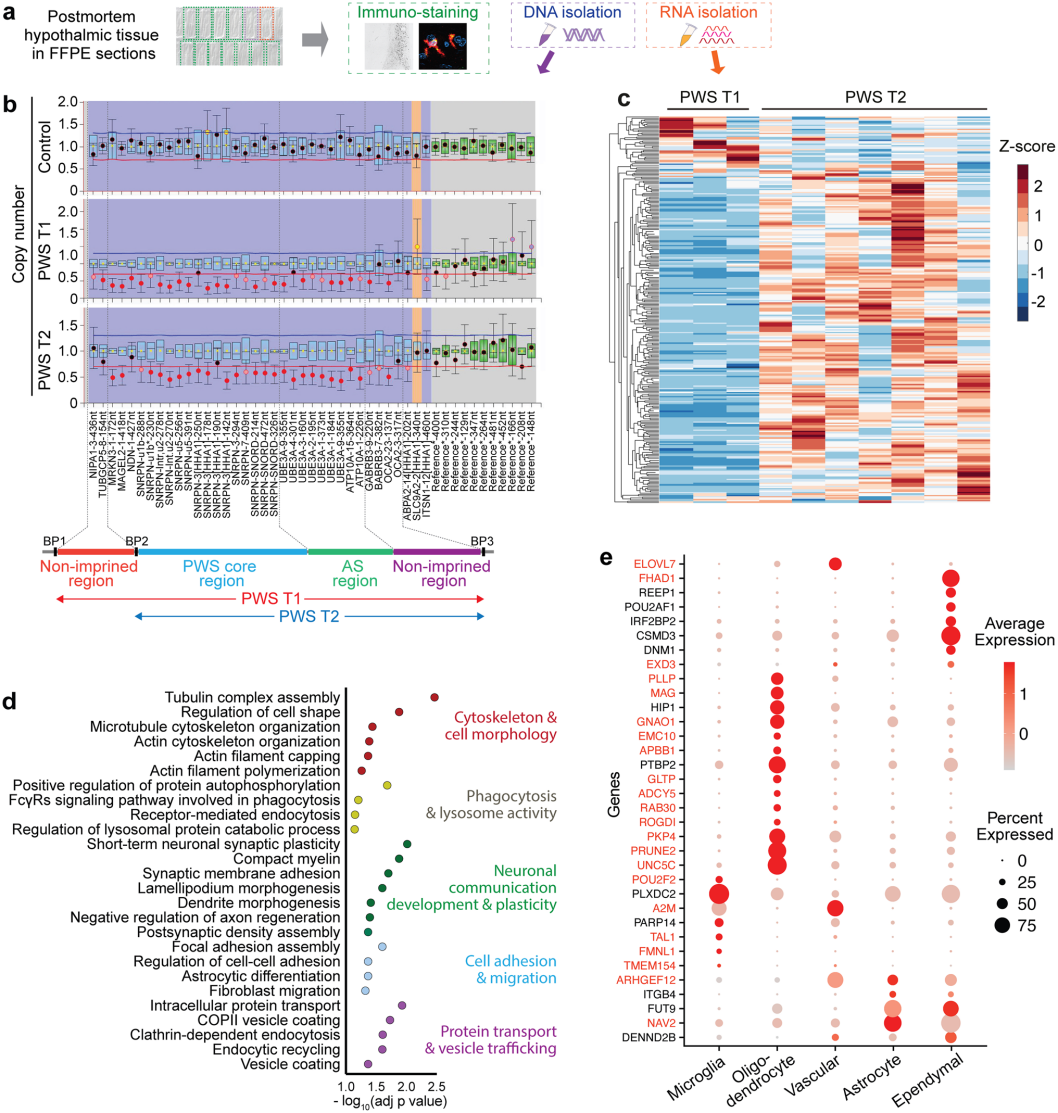
Schematic flow of the experimental setup and genetic profiling. **a** Postmortem hypothalamic tissues were formalin-fixed paraffin-embedded (FFPE) sections, and the consecutive FFPE sections were used for morphological profiling by immunohistochemistry or immunofluorescence. DNA and RNA isolated from these sections were used for genotyping and next-generation RNA sequencing. **b** Multiplex Ligation-dependent Probe Amplification (MLPA)-assisted genotyping of PWS with copy number. No deletion was observed in control subjects; PWS type 1 (PWS T1) subjects showed a 50% gene dose from BP1 to BP3 (red circles, starting from *NIPA1* and *TUBGCP5* between BP1 and BP2 (*NIPA2* and *CYFIP1* between BP1 and BP2 were not included in the MLPA analysis)), PWS type 2 (PWS T2) subjects showed a 50% gene dose from BP2 to BP3 (red circles, starting from *MKRN3* and *MAGEL2*). **c** The significant differentially expressed genes (DEGs) are depicted as within-gene Z-scores in the heatmap, representing all the genes that are significantly up- or down-regulated when comparing PWS T1 and T2. **d** The majority of the biological processes down-regulated in PWS T1 compared to PWS T2. **e** Brain non-neuronal cell-type-specific genes among the DEGs within-gene Z-score. Genes in red color are down-regulated in PWS T1 comparing to PWS T2, genes in black color are down-regulated in PWS T1 comparing to controls

To comprehensively profile both gene and protein expression using FFPE sections of postmortem brain tissues collected since 1983, we divided the sections into three distinct sets. The first set underwent Nissl staining to define anatomical orientation, and immunohistochemistry or immunofluorescence techniques were applied to visualize the expression of specific target proteins (Fig. 1a). A second set of sections was dedicated to determining PWS genetic subtypes through the MLPA assay, effectively identifying deletions in genes located on chromosome 15q11-q13 (Fig. 1b). For the third set of FFPE sections, RNA extraction was performed to facilitate subsequent bulk RNA-sequencing analysis (Fig. 1c).

The genotypic assessment allowed the categorization of subjects into three groups: Controls, PWS T1, and PWS T2. In all PWS subjects, copy number reduction was observed for genes situated on the long arm of chromosome 15q11-q13 between break point 2 (BP2) and BP3, encompassing *NECDIN, MRKN3,* and *MAGEL2*. Notably, the distinction between the two genotypes relied on the profiling of *NIPA1 and TUBGCP5* genes positioned between BP1 and BP2.

These genes exhibited normal levels in PWS T2 but displayed reduced-gene copies in PWS T1 (Fig. 1b). In contrast, control samples exhibited no alterations in copy number. Altogether, three PWS T1 and seven PWS T2 subjects were successfully identified. These PWS subjects were subsequently compared to 32 control subjects, that were matched in terms of age, postmortem delay time, and tissue fixation time (Table 1). Within the subset of three PWS T1 subjects, the body mass index (BMI) data were available for subjects aged 6 months and 4 years. Given that hyperphagia and obesity typically onset after 6 months of age, it is reasonable that the average BMI of the PWS T1 group did not display statistically significant differences when compared to the control subjects. However, as anticipated, subjects in the PWS T2 category exhibited a notably higher average BMI compared to the control group, aligning with the expected phenotype.

Delving into the transcriptomic analysis, we identified the most prominently DEGs within the context of PWS T1 deletion (Fig. 1c, Supplementary Fig. 3). Specifically, upon comparing T1 and T2, we observed a substantial downregulation of genes related to cellular integrity, including those associated with cytoskeletal structure, morphology, adhesion, migration, protein transport, and vesicle trafficking. Furthermore, we noted a decrease in the expression of genes linked to phagocytosis, lysosomal activity, as well as neuronal development and communication in PWS T1 subjects (Fig. 1d). To investigate whether these transcriptional changes were associated with specific cell types, we used publicly available processed scRNA seq data from the mammary, tuberal, and supraoptic regions of the hypothalamus from Human Brain Cell Atlas [43]. Using FindAllMarkers from the Seurat package [19], we identified cell-type-specific genes and intersected these with our DEGs [43]. This analysis revealed a strong oligodendrocyte and, to a lesser extent, microglia-specific gene profile among the DEGs that are down-regulated in PWS T1 compared to PWS T2 or controls (Fig. 1e). In addition, some of the down-regulated genes are also specifically expressed in astrocytes, vascular, or ependymal cells (Fig. 1e). Furthermore, we also discovered more than 200 neurons-enriched genes that are down-regulated in PWS T1 compared to PWS T2 or controls (Supplementary Fig. 4). Collectively, these transcriptomic signatures unveiled robust gene expression differences between PWS T1, T2 subtypes and the controls. Conjectural interpretation of these data hinted for glial and neuronal dysfunction as a pathophysiological mechanism that underlies the worsened physiological and behavioral traits in patients with a PWS T1 deletion.

### PWS T1 subjects present dysmorphic microglia

**Fig. 2:**
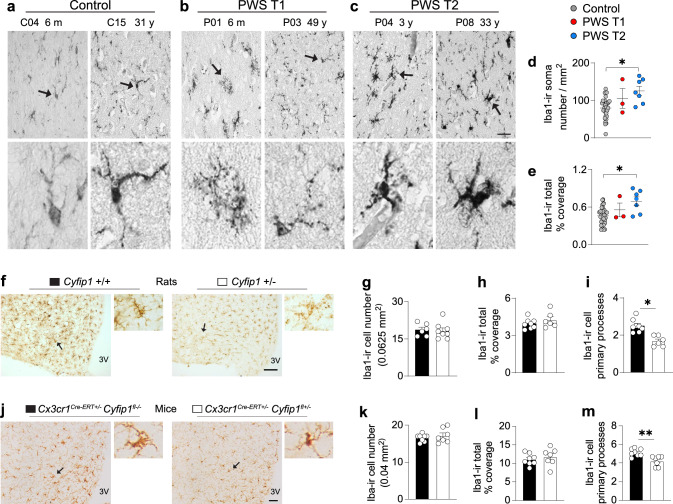
PWS T1 deletion is associated with dysmorphic microglia that are partially driven by Cyfip1 haploinsufficiency.** a**-**c** Representative images of Iba1-ir cells in the mediobasal hypothalamus of the control (n = 32), PWS T1 (n = 3), and PWS T2 (n = 7) subjects. Dark arrow-pointed microglia in the upper panel of **a** are shown at a higher magnification in the lower panel. m, months; y, years. **d, e** Comparison of the hypothalamic Iba1 soma number and relative area of coverage. **f** Immunohistochemistry for Iba1-ir microglia in the mediobasal hypothalamus of wild-type (n = 6) and Cyfip1 haploinsufficient (n = 8) male rats. Dark arrow-pointed microglia in the left panel of each genotype in **f** are shown with higher magnification in the two right panels. **g**-**i** Iba1-ir cell number and soma size and primary processes in Cyfip1+/− male rats. **j** Iba1-ir microglia in the mediobasal hypothalamus of control mice (*Cx3cr1*^*Cre−ERT*+/−^ *Cyfip1*^*fl−/−*^) (n = 8) or *Cx3cr1*^*Cre−ERT*+/−^ *Cyfip1*^*fl*^^+/−^ mice (n = 7) at the age of 32 weeks. Dark arrow-pointed microglia in the left panel of each genotype in **j** are shown at higher magnification in the right panels. Scale bar: 20 µm in **a**-**c** upper panel, 100 µm in **f**, 50 µm in **j**. Data are represented as mean ± SEM. Significance in **d** and **e** was calculated using the Kruskal–Wallis test, significance in **i** and **m** was calculated using the Student’s t-test. * p < 0.05, ** p < 0.01

Among the four haploinsufficient genes unique to PWS T1, *CYFIP1* is highly expressed in microglial cells in the brain [18, 42] and is involved in actin cytoskeleton remodelling [14]. Our transcriptomic analysis revealed pathway changes in the cytoskeleton, combined with enrichment of downregulation of DEGs in PWS T1 in comparison to PWS T2 or controls that are microglia-specific. Consequently, we first evaluated microglial morphology using Iba1-immunoreactivity (Iba1-ir). Strikingly, Iba1-ir cells in PWS T1 subjects exhibited aberrant morphology characterized by cytoplasmic deterioration and fragmentation (16.45 ± 2.8 fragments/cell), a process known as cytorhexis [48] (Fig. 2a - c). This PWS T1-associated microglial dysmorphism was not found in controls and PWS T2 subjects, in which the Iba1-ir microglia throughout the hypothalamus were morphologically intact, with few visible primary processes (Control: 1.48 ± 0.31/cell, PWS T2: 1.70 ± 0.26/cell, p = 0.62). Moreover, we observed an increased number of Iba1-ir cells and a larger relative area of coverage in the mediobasal hypothalamus of PWS T2 subjects compared to controls, indicating heightened immune activity in microglia among subjects with PWS T2 deletion (Fig. 2d, e).

Next, we investigated whether disruptive microglial morphology could be observed using other microglial functional markers. TMEM 119 and P2Y12R were used as microglial homeostatic identifiers [45]. Our findings showed that the disruption in microglial morphology observed in the Iba1-ir subset was also present in TMEM 119-ir and P2Y12R-ir microglia (Supplementary Fig. 5). However, unlike the Iba1-ir results, we did not observe any differences in the cell number or relative area of coverage between the controls and PWS T2 with TMEM 119-ir and P2Y12R-ir (Supplementary Fig. 5). Furthermore, we found that Iba1-ir microglia in the hippocampal CA1 area of PWS T1 individuals exhibited dysmorphic features similar to those observed in the hypothalamus of these subjects, with significantly lower total area of coverage (Supplementary Fig. 6).

*CYFIP1* is involved in actin cytoskeleton remodelling [14]. To determine whether the absence of *CYFIP1* can be a causal factor in microglial dysmorphism, we evaluated Iba1-ir microglial cells in the mediobasal hypothalamus of wild-type (+/+) rats and global *Cyfip1* haploinsufficient (*Cyfip1*+/−) male rats (Fig. 2f–i). We found a reduced number of primary processes due to fragmentation in the microglia of *Cyfip1*+/− rats compared to their littermates (Fig. 2i), but no significant alterations in Iba1-ir cell count or relative covered area (Fig. 2g, h). Thus, the microglial morphological disruption in global *Cyfip1* +/− rats partially recapitulates our findings in PWS T1. Of importance, microglial morphological alterations were also detected in female *Cyfip1* haploinsufficient (*Cyfip1*+/−) rats (Supplementary Fig. 7a—d). Given the robust expression of *Cyfip1* within microglial cells and the early emergence of microglial dysmorphism by the age of 6 months in PWS subjects, we generated a specialized *Cx3cr1*^*Cre−ERT*+/−^ *Cyfip1*^*fl*+/−^mouse model targeting myeloid cells, including microglia in the brain. We evaluated microglial morphology in adult (32 weeks of age) male mice. Although the number of Iba1-ir microglia and soma size in *Cx3cr1*^*Cre−ERT*+/−^ *Cyfip1*^*fl*+/−^ mice were comparable to the controls (Fig. 2k, l), the number of primary branches on each cell were profoundly reduced in the *Cx3cr1*^*Cre−ERT*+/−^ *Cyfip1*^*fl*+/−^ mice (Fig. 2m). Thus, *Cyfip1* haploinsufficiency has a detrimental impact on microglial morphology in different rodent models.

However, it is important to emphasize that in the *Cx3cr1*^*Cre−ERT*+/−^ *Cyfip1*^*fl*+/−^ mice, we did not observe cytoplasmic fragmentation in microglial cells in the brain, as was found in the brains of PWS T1 subjects. This suggests that *Cyfip1* haploinsufficiency may not be the sole driver behind the microglial dysmorphisms observed in human brains afflicted with PWS T1 deletion. This leads us to consider the possibility that other genes within the PWS T1 deletion region, such as *TUBGCP5*, which is known to be involved in microtubule dynamics [24], or common neuronal dysfunctions shared by both PWS T1 and T2 deletions, involving genes in the core PWS region, may be acting synergistically to contribute to the microglial dysmorphic changes witnessed in PWS T1 deletion brains.

### PWS Type 1 is associated with defective microglial phagolysosomal activity

**Fig. 3:**
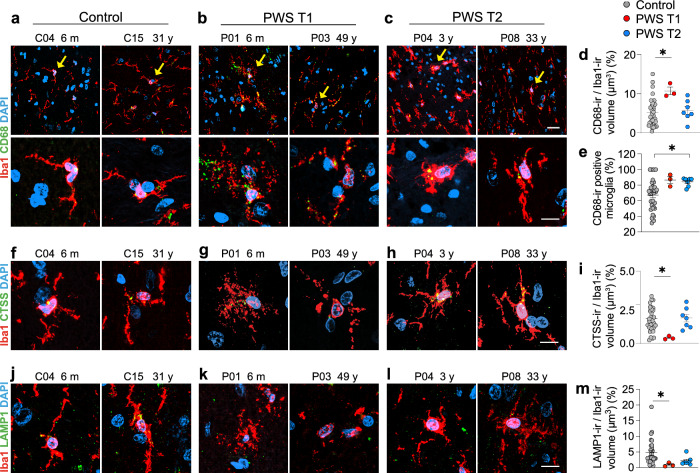
Dysmorphic microglia with PWS T1 deletion are defective in phagolysosome activity. **a**-**c** Representative images of CD68 expression in Iba1-ir microglia in the mediobasal hypothalamus of control (n = 32), PWS T1 (n = 3), and PWS T2 (n = 7) subjects. Yellow arrow-pointed microglia in the upper panel are shown at higher magnification in the lower panel of **a-c**. **d**-**e** Quantitative analysis of CD68-ir positive microglia among total Iba1-ir cells and CD68-ir volume relative to the Iba1-ir volume. **f–h** Representative images of CTSS expression in Iba1-ir microglia. **i** Quantitative analysis of CTSS-ir volume relative to Iba1-ir volume. **j-l** Representative images of LAMP1 expression in Iba1-ir microglia. **m** Quantitative analysis of LAMP1-ir volume relative to Iba1-ir volume. m, months; y, years. Scale bar: 30 µm in the upper panel of **a**-**c**, 10 µm in the lower panel of **a**-**c**, **f**–**h** and **j-l**. Data are presented as mean ± SEM. Significance was calculated using the Kruskal–Wallis test for all comparisons. * p < 0.05

The microglial immune surveillance and scavenging functions heavily depend on phagocytosis and lysosome activity [10]. Given the mechanical changes that microglial cells undergo to engulf and digest particles, we hypothesized that dysmorphic microglia in PWS T1 might exhibit significant phagocytosis defects. Therefore, we evaluated their phagocytic capacity by co-immunostaining for Iba1 and CD68, a phagosome surface marker (Fig. 3a–c). The ratio of phagosome volume to soma volume was used to assess microglial phagocytic capacity. Interestingly, we found a significantly higher CD68-ir/Iba1-ir volume ratio in PWS T1 microglia compared to controls, primarily due to an enlargement of CD68-ir phagosome particles in these cells (Fig. 3d). In contrast, PWS T2 microglia exhibited a CD68-ir/Iba1-ir volume ratio comparable to controls (Fig. 3d). However, we observed in PWS T2 a higher proportion of CD68-ir microglial cells among the total Iba1-ir microglia compared to controls (Fig. 3e), consistent with the elevated number of microglia in PWS T2 (as shown in Fig. 2d, e).

Next, we assessed whether the abnormal phagosome volume in PWS T1 was coupled with a higher capacity for debris degradation within phagolysosomes. To examine this, we investigated two lysosomal markers: CTSS, a protease belonging to the cathepsin family, and LAMP1, a cardinal lysosomal indicator marker [23, 53]. We found that, in contrast to CD68, PWS T1 microglia exhibited a significant reduction in CTSS-ir (Fig. 3f–i) and LAMP1-ir (Fig. 3j-m), indicating a decreased debris degradation capacity within lysosomes. Microglia in PWS T2 brains had CTSS-ir and LAMP1-ir levels comparable to those in the control group (Fig. 3i, m).

As *CYFIP1*-involved actin cytoskeleton remodelling plays an important role in the fusion process during the phagolysosome maturation [29, 31], we assessed the microglial phagocytic capacity in *Cx3cr1*^*Cre−ERT*+/−^ *Cyfip1*^*fl*+/−^ mice. We found a significantly higher CD68-ir/Iba1-ir volume ratio in the microglial cells of the *Cx3cr1*^*Cre−ERT*+/−^ *Cyfip1*^*fl*+/−^ mice compared to control mice (Supplementary Fig. 7e, f). These data suggest that *CYFIP1* deficiency may directly impact the phagocytic function of microglia in PWS.

### PWS T1 is associated with increased glymphatic system aquaporin 4 in the hypothalamus

**Fig. 4:**
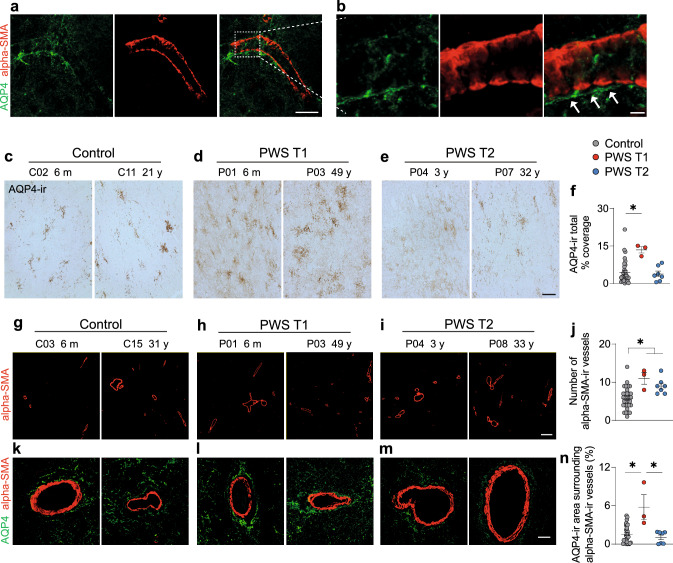
Enhanced glymphatic component aquaporin 4 expression in PWS T1 deletion. **a, b** Illustration of AQP4-ir astrocytes surrounding alpha-SMA-ir vessels that form the perivascular glymphatic system. The white dashed line-framed area in **a** is shown with higher magnification in **b**, and white arrows indicate the space between the AQP4-ir astrocytes and the alpha-SMA-ir vessel. **c-e** Representative images of AQP4-expressing astrocytes in control, PWS T1, and PWS T2 subjects. **f** Quantitative analysis of AQP4-ir covered area in the hypothalamus. **g-i** Representative images of alpha-SMA-ir vessels in control, PWS T1, and PWS T2 subjects. **j** Quantitative analysis of the number of alpha-SMA-ir vessels in the hypothalamus. **k-m** Representative images of the AQP4-ir astrocytes surrounding the alpha-SMA-ir vessels in control, PWS T1, and PWS T2 subjects. **n** Quantitative analysis of the area of AQP4-ir surrounding the alpha-SMA-ir vessels. m, months; y, years. Scale bar: 30 µm in **a**, 5 µm in **b**, 50 µm in **c**-**e,** 150 µm in **g-i**, 10 µm in **k-m**. Data are presented as mean ± SEM. Significance was calculated using the Kruskal–Wallis test for all comparisons. * p < 0.05

Given the compromised phagolysosome activity of microglia in PWS T1 brains, we hypothesized that the brain microenvironment might accumulate more cell debris and waste compared to PWS T2 brains, necessitating enhanced cleaning mechanisms. Consequently, we investigated AQP4, a critical component of the brain’s glymphatic drainage system [22, 33]. In the human brain, AQP4-ir is exclusively found in astrocytic cells, with punctate staining primarily attributed to astroglial end-feet processes, spanning the parenchymal area (Fig. 4a, b). These end-feet processes create a perivascular space, known as the glymphatic system. We detected a significantly larger AQP4-ir covered area in the hypothalamus of PWS T1 subjects compared to controls, while no significant difference was observed between PWS T2 and controls (Fig. 4c–f).

Next, we assessed hypothalamic vasculature through alpha-SMA-ir, an endothelial marker for arteries and arterioles. We found an increased number of alpha-SMA-ir vessels in PWS, irrespective of the subgenotype (Fig. 4g–i), indicating hypothalamic angiogenesis in this pathology. We further investigated the topographic association between AQP4-ir astroglia and alpha-SMA-ir vessels by evaluating the AQP4-ir surrounding the alpha-SMA vessels within a 20 µm radius from each individual vessel. We found an increased presence of AQP4-expressing astroglia in the perivascular space in PWS T1 compared to controls and PWS T2 subjects (Fig. 4k–n). These findings point towards heightened-glymphatic system activity in PWS T1 individuals requiring closer interaction with vasculature and suggesting an increased demand for the removal of harmful molecules from their brain. This response likely arises due to the defective immune scavenging and cleaning function of microglia within the brain microenvironment.

### Different PWS sub-genotype displays distinct white matter patterns

**Fig. 5:**
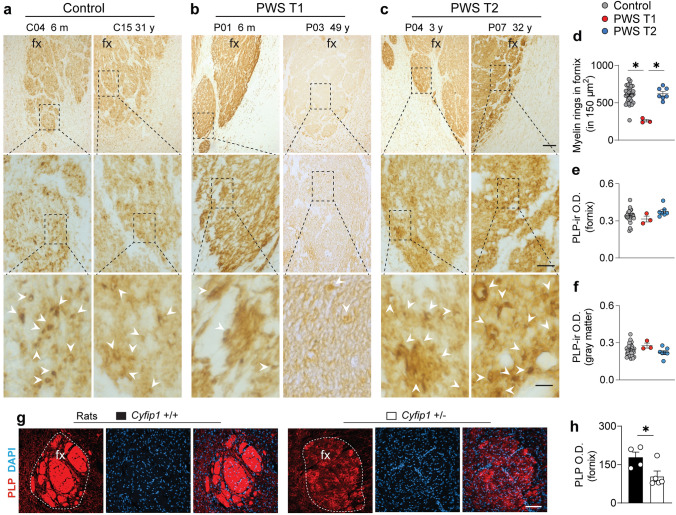
Abnormal white matter microstructure in the fornix of PWS T1 subjects. **a-c** Representative images of PLP-ir at the level of the fornix in the hypothalamus of controls (n = 32), PWS T1 (n = 3), and PWS T2 (n = 7) individuals. Framed areas in upper and middle panels are displayed in details in their lower panels respectively. Individuals with PWS T1 deletion have aberrant white matter structures, as shown by a drastic reduction in PLP-ir nodes (myelin rings, indicated by white arrowheads) throughout the fornix. **d-f** Comparison of PLP-ir myelin rings and optical density in the fornix or gray matter outside the fornix. **g** Fornix outlined by PLP-ir in the hypothalamus of wild-type (n = 4) and Cyfip1 haploinsufficient (n = 4) rats. **h** Comparison of the PLP-ir optical density in the fornix. Fx, fornix; O.D., optical density; m, months; y, years. Scale bar in **a**-**c**, 100 µm in upper panel, 20 µm in middle panel, and 5 µm in lower panel; 100 µm in **g**. Data are represented as the mean ± SEM. Significance was calculated using the Kruskal–Wallis test for **d**, **e**, and **f**. * p < 0.05

Given the downregulation of genes associated with microglia, oligodendrocytes and myelination capacity in our transcriptomic analysis, along with previous findings in *Cyfip1*+/− rats showing abnormal white matter structure—characterized by a reduced number of oligodendrocytes and decreased myelin thickness in the corpus callosum [44]—we conducted an assessment of white matter integrity in PWS brains, with a primary focus on the fornix, a major white matter tract originating from the hippocampus, passing through the hypothalamus, and ending in the hypothalamus and mammillary body [51] (Fig. 5a–c). In control and PWS T2 subjects, using PLP-ir, we observed a uniform distribution myelin ring across the fornix, suggesting normal structural and homeostatic myelination capacity. However, in PWS T1, the myelin rings were sporadic, with a significantly lower total number of myelin rings compared to controls and PWS T2 (Fig. 5d), potentially indicating the presence of underdeveloped myelin. No difference was found in the intensity of PLP-ir observed in both the fornix and the gray matter adjacent to the fornix among all the groups (Fig. 5e, f). To ascertain whether white matter deterioration in PWS T1 was exclusive to the hypothalamus, we also evaluated myelin microstructure in the anterior commissure and hippocampus. However, we did not observe structural abnormalities in any of the evaluated white matter landmarks, regardless of the PWS subgenotype (Supplementary Fig. 8 and Supplementary Fig. 9).

Microglia are closely associated with myelin sheath organization and have intimate interactions with oligodendrocytes [41]. Therefore, we examined Iba1-ir microglia within the fornix (Supplementary Fig. 10). Compared to controls (Supplementary Fig. 10a, e), microglial fragmentation was observed in the fornix of PWS T1 (Supplementary Fig. 10b, f), while an increased Iba1-ir covered area was noted in the fornix of PWS T2 (Supplementary Fig. 10c, d). Since myelin-laden microglia are a common feature of demyelinating pathologies, we investigated whether there was differential myelin phagocytosis in the PWS subtypes. However, Iba1-ir cells co-localized with PLP-ir particles were only occasionally found in microglia cell in general (Supplementary Fig. 10e–g), making it challenging to conduct a comprehensive quantitative analysis.

During myelin sheath development, the turnover of actin filaments in oligodendrocytes plays a critical role in regulating repetitive cycles of leading-edge protrusion and spreading [36]. In alignment with a previous study on *Cyfip1*+/− rats that demonstrated aberrant white matter structure [44], we found a reduction in PLP-ir in the fornix of *Cyfip1*+/− rats (Fig. 5g, h). Our data suggest a possibility that the loss of myelin rings in the fornix in the PWS T1 hypothalamus might be due to *CYFIP1* haploinsufficiency-induced actin disarrangement within oligodendrocytes.

### Both PWS T1 and T2 show hypothalamic neuropeptidergic imbalance

Functional microglia play a crucial role in supporting neural development and maintaining health. Therefore, we meticulously profiled neurons in the hypothalamus, with a focus on those producing neuropeptides that regulate energy homeostasis. Regrettably, one PWS T1 subject lacked sections for neuropeptide analysis in the infundibular nucleus and paraventricular nucleus (PVN) in the hypothalamus. We initiated our analysis at the infundibular nucleus level, particularly examining anorexigenic POMC and orexigenic NPY neurons. We noted a decrease in the POMC-ir covered area and cell count in the hypothalami of PWS T2 subjects compared to controls (Supplementary Fig. 11a–e). Interestingly, the PWS T1 infant (6 months old) exhibited increased POMC-ir parameters, while the adult did not differ from PWS T2 subjects. Due to the reduction in anorexigenic POMC-ir neurons, we also evaluated orexigenic NPY-ir neurons in the same area, finding an overall diminished number of NPY-ir cells in both PWS T1 and T2 (Supplementary Fig. 11f–j). Moving on, we examined neuronal populations within the PVN, focusing on OXT and AVP producing neurons. We found no alterations in AVP-ir neurons in the PVN of patients with PWS (Supplementary Fig. 12a–e). However, OXT-ir mirrored the pattern found in POMC-ir expressing cells, displaying a clear reduction in PWS T2 (Supplementary Fig. 12f–j). In sum, our findings confirm that dysfunction in hypothalamic neuropeptidergic machinery is a hallmark of PWS, likely driven by the PWS T2 deletion.

### PWS T1 presents worsened neural communication

**Fig. 6:**
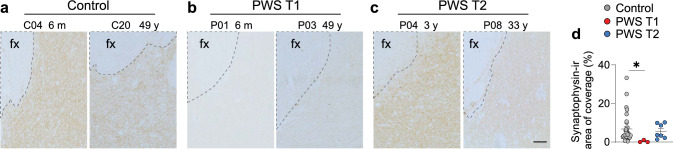
Reduced hypothalamic synaptophysin expression in PWS T1 subjects. **a-c** Representative images of synaptophysin immunoreactivity in the hypothalamus of control (n = 32), PWS T1 (n = 3), and PWS T2 individuals (n = 7). PWS T1 hypothalami showed a reduction in synaptophysin-ir compared to controls indicating defective neuron-neuron communication. Dotted lines frame the fornix. **d** Quantitative analysis of the hypothalamic synaptophysin, as demonstrated by the relative area of coverage. Fx, fornix; m, months; y, years. Scale bar: 100 µm. Data are represented as mean ± SEM. Significance was calculated using the Kruskal–Wallis test in **d**. * p < 0.05

One of the major outcomes of our transcriptome analysis is the downregulation of pathways involved in synaptic function and neuronal communication. Given the critical role of microglial scavenging and cleaning function in synaptic pruning, coupled with *Cyfip1*’s implication in synaptic homeostasis [13] and its enrichment in cortical inhibitory synaptic sites [44], we posited that PWS T1 might suffer from impaired synaptic stabilization and function. We, therefore, assessed the expression of the synaptic integrity marker synaptophysin. In controls, synaptophysin-ir exhibited a punctual pattern, evenly distributed throughout the tissue (Fig. 6a). Brains of PWS T2 did not show alterations in synaptophysin expression compared to controls (Fig. 6a, c, d). In contrast, PWS T1 brains displayed a marked reduction in synaptophysin-ir (Fig. 6b, d). Furthermore, we observed no significant changes in synaptophysin expression in the hippocampal CA1 region (Supplementary Fig. 13). Our data suggest that deleted genes in PWS T1 may contribute to the disrupted synaptic integrity in the hypothalamus.

### Confounder analysis

Considering the substantial heterogeneity in postmortem human brains, we performed a confounder analysis, considering age, tissue fixation time, postmortem delay, and BMI. The limited number of PWS T1 specimens precluded sex analysis in this group, and no sex differences were observed in the controls or PWS T2. All potential confounders were matched between the control and PWS groups, except those with data not available (see in Supplementary Table 1). Linear regression analysis revealed incidental significance for several parameters, including Iba1-ir soma number/mm^2^ vs. BMI in controls, Iba-ir relative area of coverage vs. age in PWS T1 (Supplementary Fig. 14d, e); TMEM119-ir soma number/mm^2^ and relative area of coverage vs. age in controls (Supplementary Fig. 15a, e); LAMP1-ir/Iba1-ir volume (%) vs. fixation time (Supplementary Fig. 19b); number of alpha-SMA-ir vessels vs. BMI in PWS T2 (Supplementary Fig. 21d); % of AQP4-ir surrounding the alpha-SMA vessels vs. BMI in PWS T2 (Supplementary Fig. 21e); AVP-ir soma number/mm^2^ and percentage of coverage vs. age in PWS T2 (Supplementary Fig. 27a, e); synaptophysin-ir relative area of coverage vs. fixation time in controls and PWS T2 (Supplementary Fig. 29b). However, these findings did not alter the overall implications of our study (Supplementary Figs. 14—29).

## Discussion

Although behavioral and cognitive differences have been previously documented between PWS T1 and T2 deletions, the underlying neuropathological basis has remained elusive. Our study aims to bridge this knowledge gap by unveiling distinct cellular and molecular signatures within the hypothalamus of individuals with PWS, categorized based on their T1 or T2 deletion status. Notably, we observe severe microglial abnormalities characterized by process fragmentation and disrupted soma-ramifications in PWS T1, while PWS T2 displays a distinct pattern involving inflammatory changes. In addition, we investigate white matter integrity, synaptic proteins, and neuronal communication in PWS T1, demonstrating the substantial impact of haploinsufficient gene expression. These findings provide invaluable insights into the genotype–phenotype association, enriching our understanding of the intricate neuropathological landscape in PWS.

**Fig. 7:**
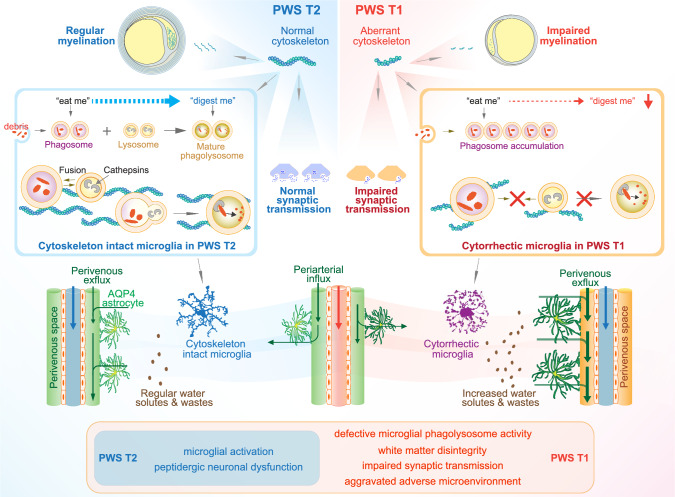
Schematic representation of the potential mechanism underlying genotype–phenotype association in patients with PWS T1 or T2 deletion. Aberrant actin cytoskeleton in PWS T1 causes cytorrhectic changes in microglia, hampers phagosome-lysosome fusion processes, causes defective myelination by oligodendrocytes, and impairs synaptic transmission. The overall disturbances in microglial immune surveillance in PWS T1 lead to the accumulation of more waste in the microenvironment and stimulate glymphatic system activity

Microglia, as the innate immune cells of the brain, play a vital role in supporting the function of neurons and other glial cells [32, 40]. Their responsibilities encompass the detection and clearance of unwanted cellular debris and waste materials, along with the production of inflammatory mediators for immune responses and defense mechanisms [25]. Previous transcriptomic studies have indicated the potential importance of microglial cells in the etiology of PWS [1]. However, the exact genotype–phenotype association in PWS has remained elusive until now. A pivotal outcome of our current study is the clarification, for the first time, of the genotype-associated phenotype related to microglial morphology and phagolysosome function. The evidence derived from both human and rodent brains strongly suggests that one of the primary cell types severely affected in PWS T1 is the microglia, with *CYFIP1* haploinsufficiency within the non-imprinted region between BP1 and BP2 playing a crucial role in driving the severity of microglial phenotypic changes. This is predominantly due to the disruptive impact of *CYFIP*1 haploinsufficiency on the actin cytoskeleton (Fig. 7), as proper phagolysosome maturation is highly reliant on the actin cytoskeleton-assisted fusion process [29, 31]. The resulting microglial cytorrhectic changes impede the fusion of phagosomes with lysosomes, leading to ineffective mature phagolysosome formation and impaired digestion of cellular debris and waste proteins (Fig. 7). Previous research has associated microglial cytorrhexis with aging-related changes in the brain and with pathological alterations in Alzheimer’s disease (AD) [47–49]. Notably, our study reveals that this morphological abnormality is already present in the early infancy brain (6 months of age) of PWS T1 patients, suggesting a sustained and severe defect in innate brain immunity throughout the lives of individuals carrying the PWS T1 deletion.

In contrast to the dysmorphic microglia observed in the brains of individuals with PWS T1, microglia in PWS T2 exhibit distinct reactive morphological changes. Prior studies from our lab and others have demonstrated that sustained microglial activation leads to hypothalamic neuronal injury and abnormal connectivity due to the production of neurotoxic and inflammatory mediators, events known to be involved in the metabolic and behavioral disruptions observed in PWS patients [28, 50, 54]. Defective post-translational modifications in neuroendocrine mediators have been documented in PWS as a whole [5, 17]. Furthermore, recent evidence emphasizes the role of Magel2, one of the core genes commonly lost in both PWS T1 and T2, in the maturation and secretion of neuropeptides [9]. This insight provides a mechanistic understanding of our observations regarding the reduced presence of neuropeptidergic neurons, a common feature in both PWS T1 and T2 subjects. Consequently, we hypothesize that the reactive microglia seen in PWS T2 are driven by the shared neuronal dysfunction associated with PWS T2 deletion, whereas the pronounced dysmorphic changes in PWS T1 microglia could arise from synergistic effects resulting from an inflammatory response combined with phagolysosome dysfunction. The question that remains is which gene(s) within the T2 deletion are responsible for the activation of microglia.

The glymphatic system, a specialized structure facilitating fluid flow between interstitial and perivascular spaces, is essential for clearing water solutes and extracellular proteins in an AQP4-dependent manner [22]. Recent evidence implicates altered glymphatic system activity in various neurological disorders, including Neuromyelitis Optica [26], type 2 diabetes mellitus [12] and AD [57]. Our study demonstrates that the increased glymphatic activity in the hypothalamus of PWS T1 subjects is associated with defective microglia. This suggests an increased demand for waste clearance in the PWS T1 brain, providing a potential therapeutic avenue (Fig. 7). The glymphatic system’s improvement through lifestyle interventions, particularly in enhancing the quality of sleep [38], might reduce the behavioral and cognitive aberrations observed in PWS T1 patients.

White matter changes have emerged as important features for the pathophysiology of PWS. Our findings indicate that white matter structural alterations in the limbic system, specifically the fornix, are associated with *CYFIP*1 haploinsufficiency in PWS T1. Since actin cytoskeleton is crucial for guiding oligodendrocyte myelination (Fig. 7), these white matter abnormalities have implications for various physiological processes, including behavioral flexibility and cognition [15]. Furthermore, recent evidence suggests that fornix microstructure is a predictor of early cognitive decline in AD [16]. Given that one of the major phenotypic expressions of PWS T1 includes worsened behavioral inflexibility and worsened cognitive performance [6, 20], our findings suggest that impaired neuronal communication mediated by the fornix between the hippocampus and hypothalamus might be one of the leading mechanisms underlying the phenotypic severity in patients with PWS T1 deletion.

It is essential to acknowledge the limitations of our study. The limited availability of hypothalamic and hippocampal tissues due to the rarity of PWS (prevalence around 1 in 10,000 to 1 in 30,000, https://www.pwsausa.org) poses a significant constraint. While our confounder analysis did not reveal significant biases in the markers in controls, the numerical limitation of PWS T1 subjects led us to pool infants and adults with PWS T1, potentially underestimating age-specific events. In addition, there was no significant difference in the BMI of controls and PWS T1 individuals. This is consistent with the nutritional phases found in PWS, where increased weight gain and abnormalities in feeding behavior typically occur at a median age older than 2 and 4.5 years, respectively [34].

The insights gained from this study carry significant implications for personalized medicine, underscoring the imperative of considering PWS sub-genotypes when devising therapeutic interventions. Specifically, addressing T1 deletion necessitates focused efforts on restoring microglial function and bolstering microglial phagolysosome activity. The fact that microglia originate from myeloid cells offers an intriguing possibility: recent research showcased the feasibility of replacing defective brain microglia through systemic hematopoietic cell transplantation, followed by targeted enhancement of microglial replacement, as demonstrated in a mutant mouse model of Alzheimer’s disease [55]. This paves the way for a potential therapeutic strategy for PWS T1 patients. Early diagnosis enables the transplantation of healthy donor bone marrow at a young age, potentially ensuring sustained, relatively normal microglial function throughout the individual’s lifetime. In contrast, for patients with a T2 deletion, managing inflammation while preserving microglial phagolysosome function emerges as a pivotal strategy for addressing the neuropathological effects in these patients.

### Supplementary Information

Below is the link to the electronic supplementary material.

Supplementary file 1 (PDF 8450 kb)

Supplementary file 2 (XLSX 193 kb)

## Data Availability

All data associated with this study are present in the paper or the Supplementary Materials. Any additional information required to reanalyze the data reported here is available from the corresponding authors upon reasonable request.
