# Supplementary material for: Microglial phagolysosome dysfunction and altered neural communication amplify phenotypic severity in Prader-Willi Syndrome with larger deletion
Source: Acta Neuropathol. 2024 Mar 31;147(1):64. doi: 10.1007/s00401-024-02714-0 (PMC10982101; doi:10.1007/s00401-024-02714-0)
Supplement: Supplementary file 1 — (PDF 8450 kb) [file 401_2024_2714_MOESM1_ESM.pdf]

Electronic Supplementary Material

Microglial phagolysosome dysfunction and altered neural communication amplify phenotypic severity in Prader-Willi Syndrome with larger deletion

Supplementary materials and methods

Histology

All the formalin-fixed paraffin-embedded (FFPE) postmortem hypothalamic and hippocampal tissues were coronally serially sectioned from rostral to caudal at 6 µm. The anatomical orientation of the infundibular nucleus and paraventricular nucleus of the hypothalamus was determined by Nissl staining and confirmed by the presence of neuropeptide Y-immunoreactive (NPY-ir) or oxytocin-ir neurons, respectively. For histological procedures, the sections were mounted on glass slides (Superfrost+) and dried on a 37°C heating plate. After 48 h, the sections were deparaffinized in 100% xylene, rehydrated in graded ethanol (100% – 50%), and rinsed in distilled water.

For studies with rodents, animals underwent anaesthesia followed by transcardial perfusion with ice-cold PBS followed by fixation with 4% paraformaldehyde. Brains were harvested and post-fixed by immersion in a solution of 4% paraformaldehyde for 24 h. The brains were washed with PBS and transferred to 30% sucrose solution for cryoprotection. Tissue was considered optimal for use for cryo-sectioning when it sank to the bottom of the bottle. The brains were mounted in optimal cutting temperature compound embedding medium, frozen at -20°C, sectioned (30 µm thickness), and collected in a cryoprotectant solution, in which they were stored until use.

Genotyping

Genotyping of postmortem human brain tissue

The PWS sub-genotype was determined using a Multiplex Ligation-dependent Probe Amplification (MLPA) assay commercially designed for targeting Prader-Willi Syndrome/Angelman Syndrome (ME028-100R, MCR Holland). Briefly, FFPE sections underwent a step-by-step immersion protocol of rehydration based on xylene-ethanol-TBS. The samples were then heated and incubated overnight in proteinase K, which promoted the overall digestion of proteins, whereas all nucleic acids, including DNA and RNA, remained intact. The next day, the samples were incubated for 1 min at 95°C and hybridized with the probes for 16 h at 60°C. The following day, ligation of the hybridized probes and PCR amplification of the ligated probes were performed according to the manufacturer's instructions.

The following genes were used to determine the genotype by evaluating copy numbers and methylation patterns: *NIPA1* (T1), *TUBGCP5* (T1), *MRKN3* (T1 and T2), *MAGEL2* (T1 and T2), *NECDIN* (T1 and T2), exons on *SNRPN* regions (T1 and T2), *UBE3A* (Angelman Syndrome), and genes located in the 15q11-13 flanked by *ATP10A*, *GABRB3*, *OCA2*, *ABPA2*, *SLC9A2*, and *ITSN1*. Additional reference genes were used as provided by the manufacturer. The reference sequences for the transcripts can be found on the manufacturer's website (www.mcrholland.com). Data analysis was performed using Coffalyser.Net.

Genotyping of Cyfip1 heterozygous knockout mice

Genotyping was conducted using mouse ear lobe tissues, according to the protocol provided by GemPharmatech. For PCR to detect the *Cyfip1* delta band after Cre mediated recombination, sequences of primers were set as follows: Forward: 5'- CCATTGCCAGATTTCTAGTCACATC-3'; Reverse: 5'- CATGCTCATAGCTTCTTCTCCAGC-3'. The WT band was approximately 274bp the delta band was 378bp.

RNA isolation, sequencing and data analysis

To isolate RNA from FFPE human hypothalamic tissues, FFPE sections were placed in sterile Eppendorf tubes and rehydrated using xylene and ethanol immersion (100%, twice). The sections were thoroughly vortexed and centrifuged for 5 min at 14000 RPM at room temperature in between steps. After the final ethanol step, samples were air-dried for 10-15 minutes. Next, approximately 150 µL of PKD buffer (Qiagen) was added to the tube, followed by 10 µL of proteinase K (both for tissue and protein digestion). The Eppendorf tubes were incubated at 56°C for 15 min with constant shaking at 700 rpm. Followed by an additional shaking step at 80°C with continuous shaking. The samples were then immediately transferred to ice for 3 minutes; then centrifuged for 15 minutes at 13’500 rpm at room temperature. The supernatant was transferred to a new tube and 16 µl of DNase Booster Buffer (Qiagen) was added plus 10 µL DNase I stock solution (Qiagen), and incubated at room temperature for 15 min. Samples were then subjected to RNeasy FFPE Kit instructions (Qiagen, #73504) eluted in 14 µl RNase-free water. RNA concentration was measured using Bioanalyzer RNA Pico Chips. In terms of RNA quality, we did not observe a clear difference associated with tissue age or postmortem delay. Samples exhibiting poor RNA concentration or a low 260/280 ratio (RNA was isolated from comparable tissues) were excluded from the RNAseq study. Due to this exclusion of samples, two additional samples from control subjects were incorporated into the RNAseq study. These two subjects were not assessed using morphological parameters. Consequently, cDNA libraries were constructed for next-generation RNAseq analysis on an Illumina sequencing platform.

Immunohistochemistry and Immunofluorescence staining

Immunohistochemistry and immunofluorescence staining were conducted by our standard procedures for postmortem human brain tissue. In brief, after deparaffinization in xylene and rehydration through graded ethanol, FFPE sections were washed in Tris-buffered saline (TBS, 0.05 M Tris, 0.15 M NaCl, pH7.6). Heat-induced epitope retrieval using microwave treatment (10 min 700 W) was performed with Tris citrate buffer (pH 6.0) or Tris buffer (pH 9.0) according to pilot studies (data not shown). After cooling, the sections were treated with 3% hydrogen peroxide in SUMI incubation buffer (0.25% gelatin and 0.5% Triton X-100 in TBS (pH 7.6)) for 10 min. Sections were then washed in TBS, incubated with primary antibodies for one hour at room temperature, and incubated overnight at 4°C. Primary antibodies against NPY and POMC were diluted in SUMI-milk buffer (5% non-fat milk), and for CD68/Iba1, CTSS/Iba1, LAMP1/Iba1, PLP/Iba1 or AQP4/alpha-SMA co-staining, the primary antibodies were diluted in SUMI-normal goat serum (10%) (G6767, Merck). The next day, sections were rinsed. For immunohistochemistry of Iba1-ir, TMEM119-ir, P2Y12R-ir, NPY-ir, POMC-ir, AVP-ir, or oxytocin-ir, sections were incubated with biotinylated

secondary antibody respectively, and the product was visualized by incubation in 0.5 mg/mL 3,3'-diaminobenzidine (DAB) (Sigma Chemical Co.) in TBS containing 0.01% H_2O_2 and 0.2% ammonium nickel sulphate (ABH; Brunschwig). Developments of PLP-ir, synaptophysin-ir, or AQP4-ir were performed using DAB without nickel sulphate. For each immunofluorescence co-staining, biotinylated secondary antibody against one of the primary antibodies were incubated, followed by incubation with a fluorophore-conjugated secondary antibody against the other primary antibody together with a fluorophore-conjugated Streptavidin. DAPI nuclei-counterstaining was selectively performed. The primary and secondary antibodies are listed in Supplementary Table 2. Iba1-ir detection in free-floating sections of Cyfip1 +/+ or Cyfip1+/- rat brains, and in *Cx3cr1*$^{Cre\text{-}ERT+/-}$ *Cyfip1*$^{fl+/-}$ or control (*Cx3cr1*$^{Cre\text{-}ERT+/-}$ *Cyfip1*$^{fl-/-}$) mouse brains, CD68/Iba1 detection in *Cx3cr1*$^{Cre\text{-}ERT+/-}$ *Cyfip1*$^{fl+/-}$ or control mouse brains were performed in a similar manner.

Images acquisition and quantitative analysis

Immunohistochemically stained images were captured with a SONY black and white camera and/or an Axio Scanner (ZEISS) and analyzed using FIJI and/or QuPath software. Images from the immunofluorescence staining were acquired using a Leica SP8-SMD confocal microscope. Blinded assessment was employed during the analysis of all images to prevent any subjective influences. Quantification of POMC-ir, NPY-ir, oxytocin-ir, and AVP-ir neurons was performed by manually outlining the area of interest based on the location of the positive signal. Depending on tissue availability usually 2-3 stained sections were available. Patient 2002-030 (PWS T1) did not have tissue available that allowed the analysis of neuropeptides in the infundibular nucleus and paraventricular nucleus and was therefore excluded from neuropeptide staining. Subsequently, the "particle analysis" software tool was used to determine the number and area of coverage of the positive signal in the outlined area. Particles between 30 µm^2 and 300 µm^2 were considered soma of neurons based on pilot studies. The total soma number was divided by the area of the outline, resulting in the soma number/mm^2. The percentage of the outlined area occupied by immunoreactive positive particles was calculated as the percentage area masked. A similar strategy was employed for microglial markers (Iba1-ir, TMEM 119-ir, and P2Y12R-ir). In sections adjacent to those used for the neuronal markers, the number of microglia cells was determined using a pre-established (and constant) outline generated, mirroring the neuronal distribution pattern, thus covering the mediobasal hypothalamus. Positive particles larger than 20 µm^2 and smaller than 100 µm^2 (size was determined in the pilot study) were considered a positive soma of microglia cells, which allowed the calculation of soma/mm^2 and the relative percentage of coverage. Since PWS T1 subjects presented dysmorphic microglia, the calculations for this parameter were performed only on cells that presented nuclear counterstaining, to guarantee that only cells were being taken into account. A similar strategy was employed in sections from rodent brains covering the arcuate nucleus area. Synaptophysin-ir and AQP4-ir relative areas of coverage were quantified by the determined threshold of positive particles generating a masked representation of both staining in a predetermined framed area using the "pixel classification" tool in QuPath. The relative coverage of the mask in relation to the total framed area was determined and used for comparison between the groups. PLP-ir was analyzed through the "intensity features" and the average optical density of PLP in white matter shreds was used as a read out. PLP-ir myelin rings

were quantified manually in a fixed outlined area (150 μm^2) by two independent experienced researchers with blinded assessment. Three 150 μm^2 regions of interest in the same section were then quantified and averaged by subject.

For immunofluorescence staining, three to five fields were used for each marker (co-labelling of CD68/Iba1, CTSS/Iba1, LAMP1/Iba1, PLP/Iba1, AQP4/alpha-SMA) and subsequent quantification. Images were obtained in 32-bit sequential standard mode at 200 Hz in a 1024 × 1024 format. The laser intensity was constant within the image acquisition of the same marker and was determined in a pilot acquisition. Analysis of the 3D volumes of the deconvolved images was performed using the Imaris 9.0. For CD68, CTSS and LAMP1 volume analysis within Iba1-ir microglial cells, CD68-ir, CTSS-ir, and LAMP1-ir particles (i.e. phagolysosomes) were analyzed in individual cells and averaged by subject. Only cells that were positive for the aforementioned markers were taken in consideration in the quantification, and 2-6 cells per field were analyzed.

Specifically, the analysis for the relative AQP4-ir surround the alpha-SMA-ir vessels was performed using QuPath software. In brief, a mask for the signal of alpha-SMA-ir and AQP4-ir particles was created using the "pixel classification" tool. No minimum particle size was imposed for either marker. Next, a circular area with a radius of 20 μm was created for every vessel annotation using the "expand annotation" tool. Then, a masked signal for AQP4-ir was created, but no minimum particle area or hole size was imposed. The area of AQP4-ir signal within every expanded object in three distinct fields was calculated, transformed into a percentage in relation to the total area of the expanded annotation, and averaged by subject.

Figure preparation was performed using the Adobe Illustrator software (Adobe Systems Inc., San Jose, CA, USA). Color correction was occasionally performed when individual pictures were assembled to figure panels for publication. No specific features within the image were enhanced, obscured, introduced, moved, or removed.

Supplementary Table 1: Clinicopathological information of control and PWS subjects

Subject	Sex	Age (years)	BMI	PMD (hours)	FT (days)	Cause of death and Clinical diagnosis
**Controls**						
C01	F	0,1	/	24	62	Perinatal asphyxia following utero-placental accident
C02	M	0,4	/	66	238	Sudden Infant Death Syndrome
C03	M	0,5	19	10	221	Sudden Infant Death Syndrome
C04	F	0,5	/	17	28	Cardiomyopathy
C05	F	0,7	16	13	238	Sudden Infant Death Syndrome
C06	M	2	14	40	80	Severe post operative shock lung; hepatoblastoma
C07	M	2	/	/	210	Dehydration
C08	M	2	/	41	41	Cerebral anoxia, hypovolemic shock, dehydration
C09	M	6	/	3,5	41	Intestinal necrosis and peritonitis
C10	M	20	25	8	82	Heart failure and acute hemorrhagic pneumonia
C11	F	21	/	20	/	Myocardial infarct
C12	M	22	/	17	27	Hypertrophic cardiomyopathy
C13	M	23	/	65	34	Heart attack
C14	F	26	41	87	26	Respiratory arrest. Tonsillar hypertrophy; epilepsy. Eating disorder
C15	M	31	23	34	35	Necrotic pancreatic tumor
C16	F	33	/	23	65	Anoxia; asthmatic bronchitis
C17	M	35	/	14	214	Respiratory insufficiency, pulmonary aspergillosis
C18	F	35	24	56	/	Respiratory insufficiency secondary to mammary cancer metastasis
C19	F	43	18	50	/	Metastasis of a renal gland carcinoma.
C20	M	49	20	22,3	33	Colon carcinoma with metastasis.
C21	M	49	24	6,3	54	Euthanasia. Hodgkin's lymphoma
C22	F	49	25	13	165	Respiratory insufficiency. Metastasized cervix carcinoma
C23	F	50	19	41	72	Euthanasia. Metastasized Pancoast tumor
C24	F	50	23	53	27	Coma. Bronchopneumonia
C25	M	54	19	8	59	Respiratory insufficiency & bronchopneumonia, hepatocellular carcinoma
C26	F	60	23	8	87	Ovarium carcinoma
C27	F	65	20	9,5	10	Cardiac failure. Mamma tumor
C28	M	56	30	5,5	35	Cardiac infarction
C29	F	71	33	7,2	53	Renal insufficiency by hypertensive nephropathy
C30*	M	73	31	4,4	60	Pneumonia
C31	F	76	32	14,5	27	Plaque hemorrhage and thrombosis in the coronary artery and circumflex
C32	F	77	33	3	39	Pulmonary metastasis of cell vulva carcinoma
C33*	M	83	23	8,8	65	Pneumonia
C34*	F	91	18	4,3	64	Heart infarction
C35#	M	59	24	65,8	180	Massive lung embolism. Peripheral and central vascular disorder.
C36#	M	63	25	32,3	35	Myocardial infraction. Cardiac failure.
**PWS T1**						
P01 (T1)	F	0,5	17	9,8	60	Positional asphyxia (patient found dead in bed, lying face down)
P02 (T1)	F	4	39	/	157	Apnea/asystole
P03 (T1)	M	49	/	/	/	Cause of death unknown. Diabetes
**PWS T2**						
P04 (T2)	M	3	/	41	63	Cause of death unknown
P05 (T2)	F	25	22	35	26	Cardiac arrest during an operation
P06 (T2)	F	30	52	4,8	/	Dyspnea, hypotension, and acidosis
P07 (T2)	M	32	/	48	59	Sudden death after fever and diarrhea
P08 (T2)	F	33	71	5	31	Anuria and hypotension
P09 (T2)	M	50	25	/	/	Acute heart failure
P10 (T2)	F	67	26	6,5	46	Pulmonary infection

FT, fixation time; PMD, postmortem delay; /, data unavailable. * These three subjects were only studied with their hippocampal tissues, their anthropometric data were not included in data analysis. # These two subjects were only used for RNA sequencing study.

Supplementary Table 2: List of antibodies for immunohistochemistry and immunofluorescence

Protein	Source	Host	Catalog number	PMID	Dilution	Antigen retrieval
Proopiomelanocortin	Phoenix Pharmaceutics	Rb	27-52	32814716	1 / 1000	pH 6.0
Neuropeptide Y	NIN	Rb	Niepke 261188	11836343, 1385516	1 / 1000	
Arginine-vasopressin	NIN	Rb	Truss 86, C.P.230686	3243229, 1100756	1 / 1000	
Oxytocin	Abcam	Rb	ab2078	28415720	1 / 15000	
Ionized calcium-binding adapter molecule 1	Synaptic Systems	Rb	234003	32814716	1 / 200	pH 6.0
Ionized calcium-binding adapter molecule 1	Wako	Rb	019-19741	33101272	1 / 1000	
Ionized calcium-binding adapter molecule 1 (for proteolipid protein co-staining)	Abcam	Gt	ab107159	32355963	1 / 150	pH 6.0
Transmembrane Protein 119	Abcam	Rb	ab209064	32814716	1 / 600	pH 6.0
P2Y purinoceptor 12	Atlas antibodies	Rb	HPA014518	35808999	1 / 2000	pH 6.0
Cluster of differentiation 68 (human)	Agilent	Ms	M0814 (clone KP1)	36497449	1 / 400	pH 6.0
Cluster of differentiation 68 (mouse)	Abcam	Rat	Ab53444	28954222	1 / 500	
Cathepsin S	Santa Cruz Biotechnology	MS	sc-271619	33480093	1 / 200	pH 9.0
Proteolipid protein	Abcam	Rb	ab254363	35344427	1 / 1000	pH 6.0
Synaptophysin	Agilent	Ms	M7315 (SYNAP)	21658579	1 / 200	pH 6.0
Aquaporin 4	Atlas Antibodies	Rb	HPA014784	27893874	1 / 5000	
Alpha-smooth muscle actin	Sigma Aldrich	Ms	A5228	37400893	1 / 1000	pH 6.0
Lysosomal-associated membrane protein 1	Atlas antibodies	Ms	AMAb91299		1 / 1000	pH 6.0
anti-Rb biotinylated	Vector Laboratories	Hs	BA-1100		1 / 400	
anti-Ms biotinylated	Vector Laboratories	Gt	BA-9200		1 / 400	
anti-rat biotinylated	Vector Laboratories	Gt	BA-9400		1 / 400	
VECTASTAIN® ABC Kit	Vector Laboratories		PK-6100		1 / 800	
anti-Rb 594	Invitrogen	Gt	A11037		1 / 400	
Streptavidin 488	Invitrogen		S32354		1 / 400	

NIN, Netherlands Institute for Neuroscience; Rb, rabbit; Ms, mouse; Gt, goat; Hs, horse.

Supplementary Table 3: Detailed statistics for the quality of the RNA sequencing data.

Sample	Raw reads	Raw data (G)	Effective (%)	Error (%)	Q20 (%)	Q30 (%)	GC (%)
P01	48686596	7302989400	60.34	0.03	96.24	91.89	53.05
P02	52799434	7919915100	75.39	0.02	97.83	94.45	51.69
P03	56818190	8522728500	78.85	0.02	98.40	95.31	51.70
P04	43301166	6495174900	55.05	0.03	95.97	91.80	53.43
P05	58547562	8782134300	68.67	0.03	96.55	92.38	49.81
P06	54104764	8115714600	49.34	0.03	95.79	91.71	54.63
P07	40870888	6130633200	65.35	0.03	97.26	93.47	54.23
P08	59580220	8937033000	64.45	0.03	97.03	93.67	52.88
P09	47431810	7114771500	59.80	0.03	96.65	93.22	55.94
P10	64278742	9641811300	67.67	0.03	97.77	94.50	54.64
C02	48009626	7201443900	61.16	0.03	96.54	92.73	54.47
C03	52800706	7920105900	69.74	0.03	97.23	93.61	53.12
C04	62353912	9353086800	63.31	0.02	97.98	94.99	53.74
C05	58098042	8714706300	61.92	0.03	96.20	92.46	53.99
C06	60939180	9140877000	71.62	0.03	97.73	94.32	53.72
C08	71241940	1,0686E+10	54.49	0.03	95.79	91.65	54.21
C09	48612142	7291821300	68.04	0.03	96.81	93.04	54.75
C10	40340002	6051000300	52.04	0.03	95.97	91.91	54.46
C11	67823806	1,0174E+10	55.65	0.03	96.70	92.90	54.19
C12	47556194	7133429100	50.41	0.03	95.56	91.43	53.74
C13	57019740	8552961000	65.49	0.03	95.73	91.68	52.58
C15	44809770	6721465500	52.03	0.03	95.29	91.05	54.34
C16	55808338	8371250700	67.95	0.03	97.30	93.79	53.27
C25	64255764	9638364600	75.73	0.02	98.14	95.00	54.92
C27	58051406	8707710900	55.83	0.03	96.18	92.14	54.67
C35	83609174	1,2541E+10	82.23	0.02	97.99	94.80	55.35
C36	77246944	1,1587E+10	72.75	0.03	96.59	92.63	54.96

Raw reads: total amount of reads of raw data. Raw data: (Raw reads) * (sequence length), calculating in G. Effective: (Clean reads/Raw reads) * 100%. Error: base error rate. Q20, Q30: (Base count of Phred value > 20 or 30) / (Total base count). GC: (G & C base count) / (Total base count).

Supplementary Figures

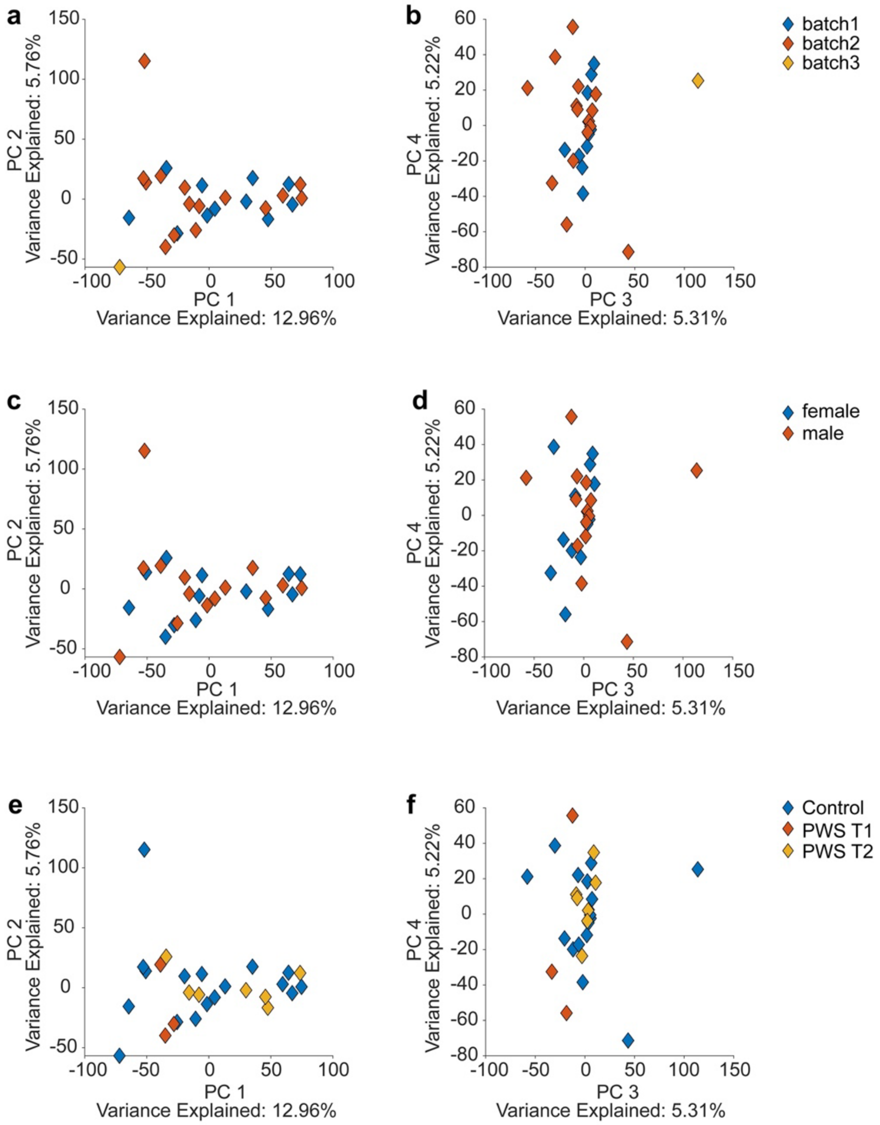

Supplementary Fig. 1

Principal-component (PC) analysis test for potential bias in the data by batch (of RNA isolation) (a, b), sex (c, d), and genotype group (e, f) as cofactors. No conspicuous source of bias could be identified.

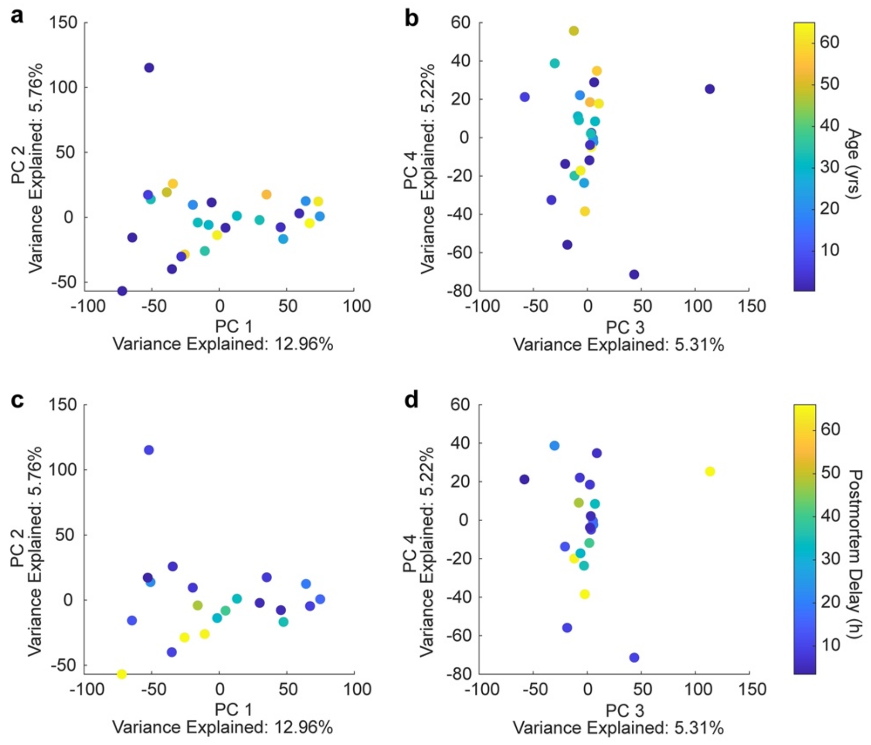

Supplementary Fig. 2

Principal-component (PC) analysis test for potential bias in the data by age (a, b) and postmortem delay (c, d) as cofactors. No conspicuous source of bias could be identified.

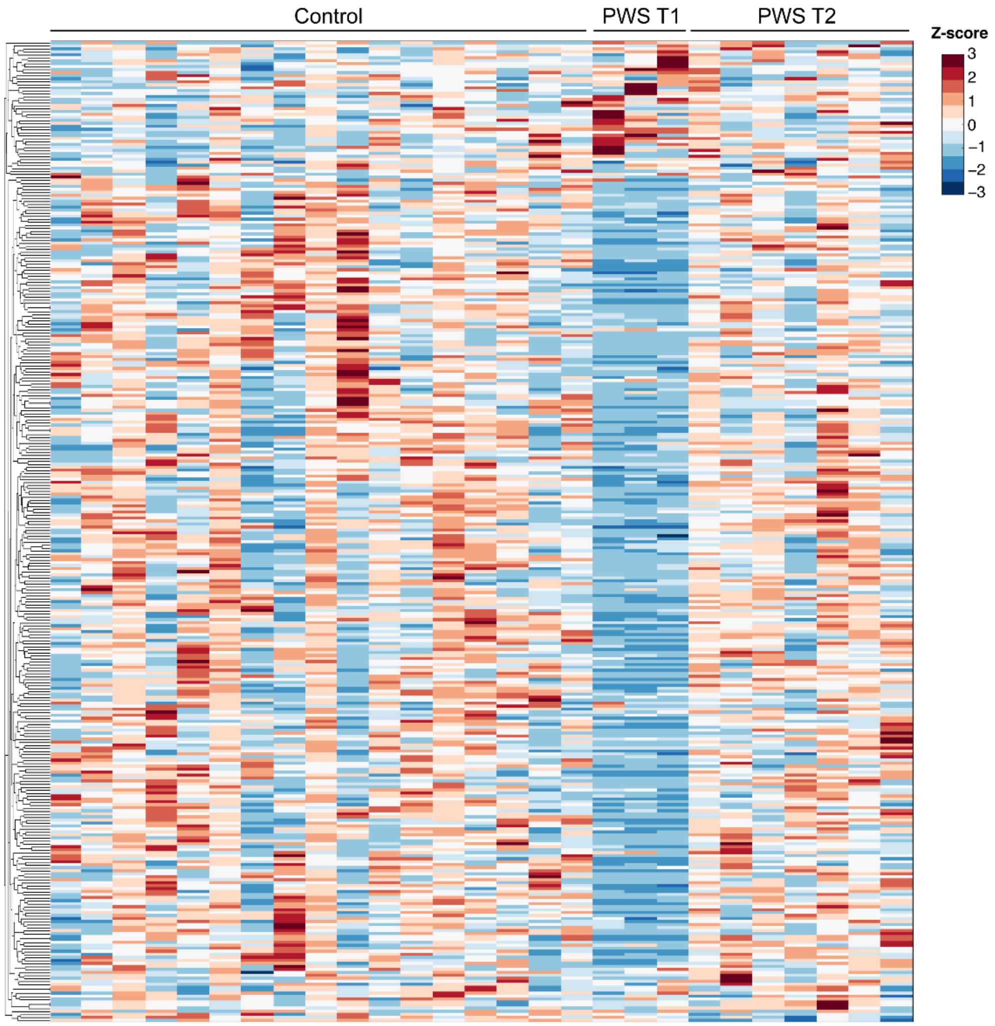

Supplementary Fig. 3

Heatmap representing the significant DEGs between controls, PWS T1, and PWS T2 shown as within-gene Z score.

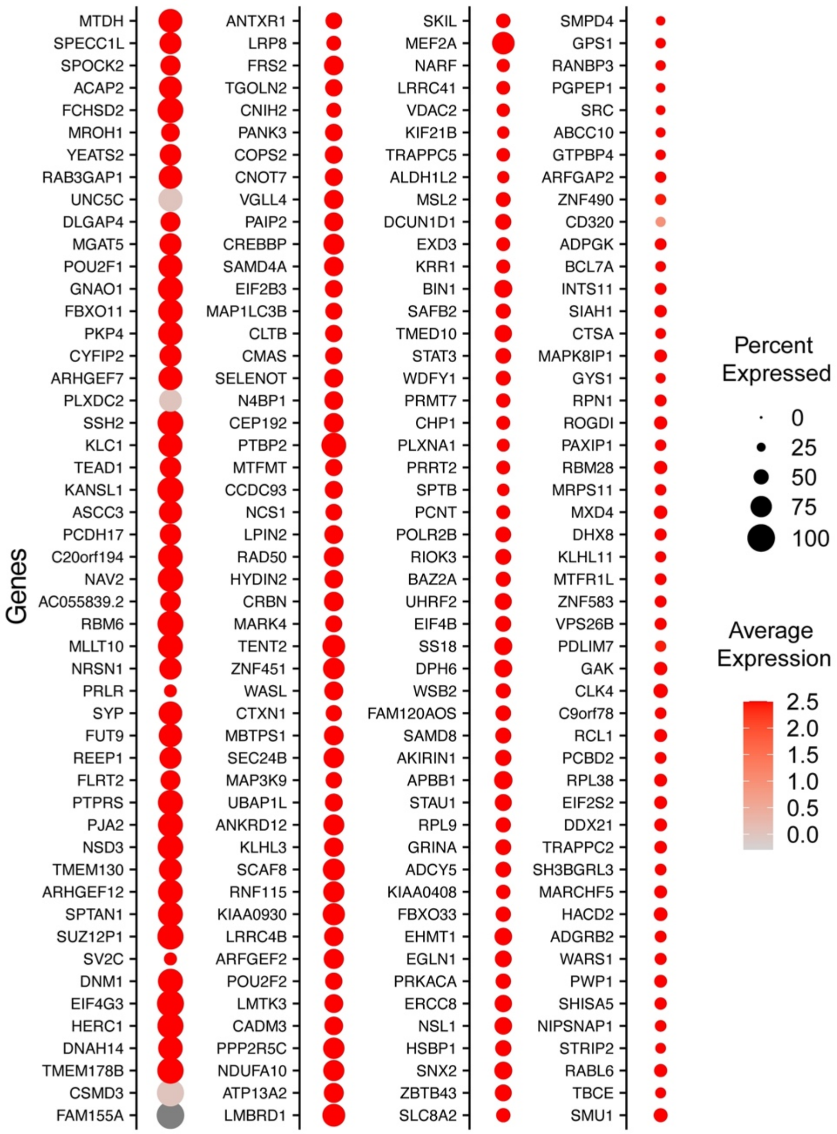

Supplementary Fig. 4

Brain neuron-enrich genes among the DEGs within-gene Z-score.

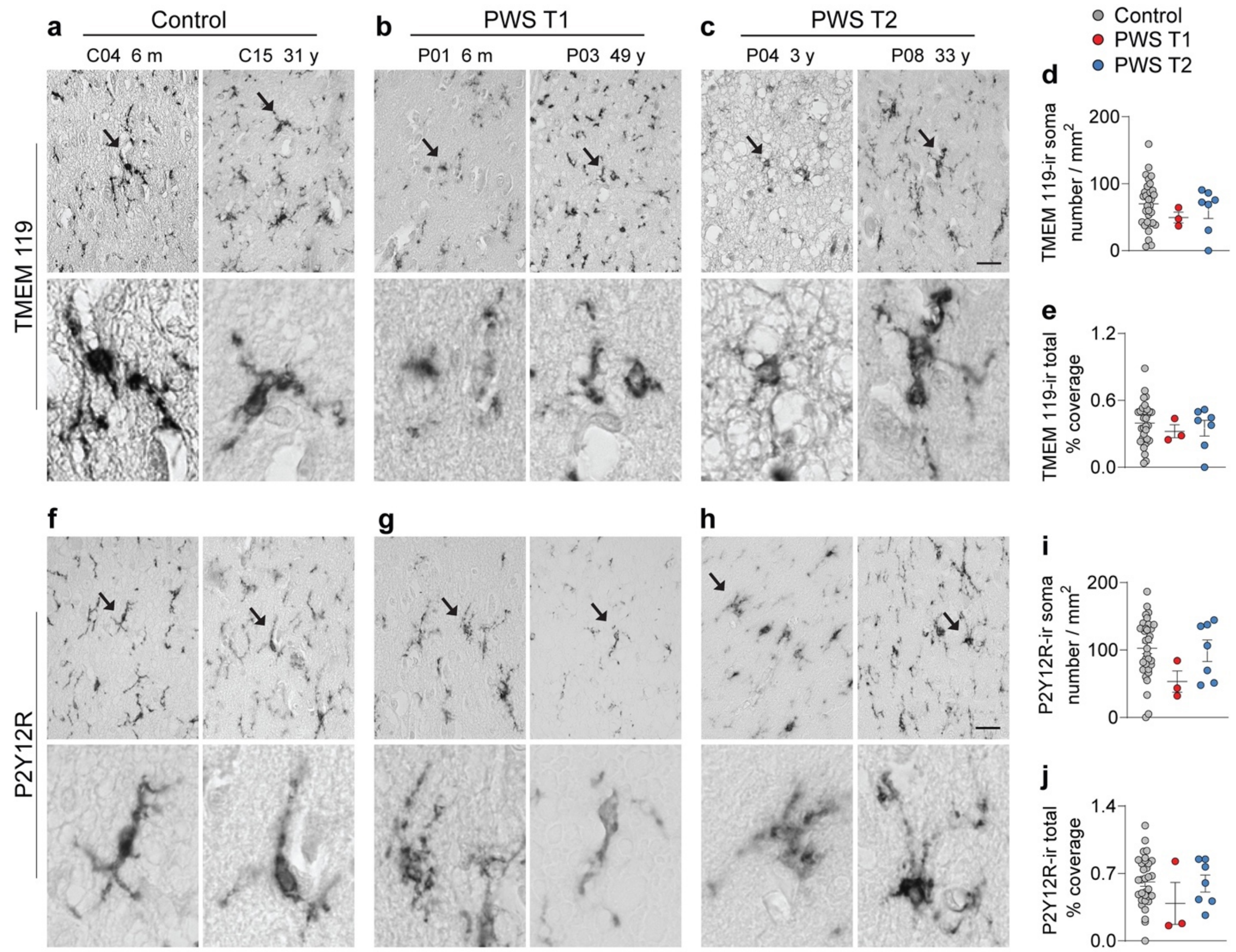

Supplementary Fig. 5

Morphological profiling of microglia by the microglia-specific homeostatic marker TMEM 119 and P2Y12R. a-c Representative images of TMEM-119-ir cells in the hypothalamus of control (n=32), PWS T1 (n=3), PWS T2 (n=7) subjects. Cells indicated by arrows in the upper panel are shown in the lower panel. TMEM-119-ir indicates intact cell morphology in the hypothalamus of control and PWS T2 subjects. In contrast, PWS T1 TMEM-119-ir cells present a disconnected soma and processes, which are fragmented, as observed in Iba1-ir cells. **d**, **e** Comparison of TMEM-119-ir soma number/mm^2 and relative area of coverage. **f-h** Representative images of hypothalamic P2Y12R-ir cells from control (n=32), PWS T1 (n=3) and PWS T2 (n=7) subjects. Cells indicated with arrows are shown at higher magnification in the lower panels. The aberrant morphology observed in different subsets of microglia in PWS T1 was also evident in P2Y12R-ir cells. **i**, **j** Quantitative analysis of P2Y12R-ir soma number/mm^2 and the relative area of coverage. m, months; y, years. Scale bar: 20 µm in **a-c** and **f-g** in the upper panel. Data are represented as mean ± SEM.

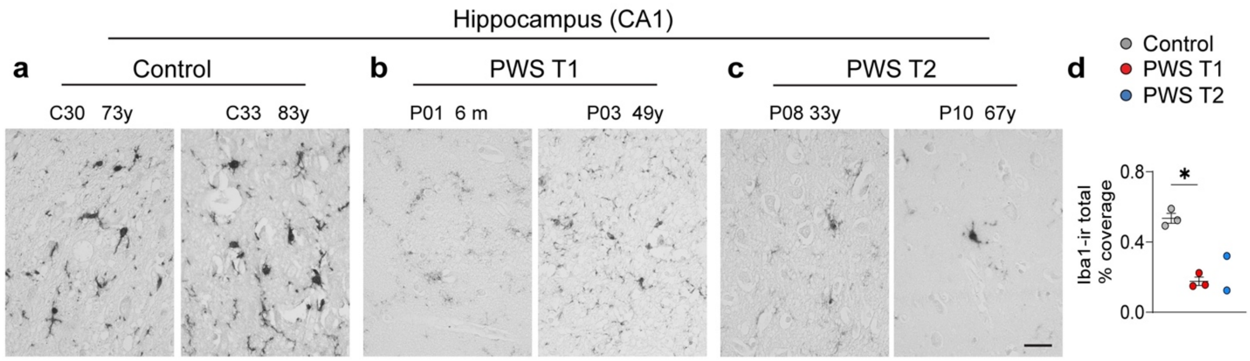

Supplementary Fig. 6

PWS T1 deletion is associated with dysmorphic microglia in the hippocampus. a-**c** Representative images of Iba1-ir cells in the hippocampal CA1 area of control (n=3), PWS T1 (n=3), and PWS T2 (n=2) subjects. Note that fragmented Iba1-ir was found exclusively in the hippocampus of PWS T1 subjects, similar to the findings in the hypothalami of these subjects. **d** Comparison of hippocampal Iba1-ir relative area of coverage. m, months; y, years. Scale bar 20 µm in **a**-**c**. Data are presented as mean ± SEM. Significance was calculated using the Kruskal–Wallis test. * $p < 0.05$.

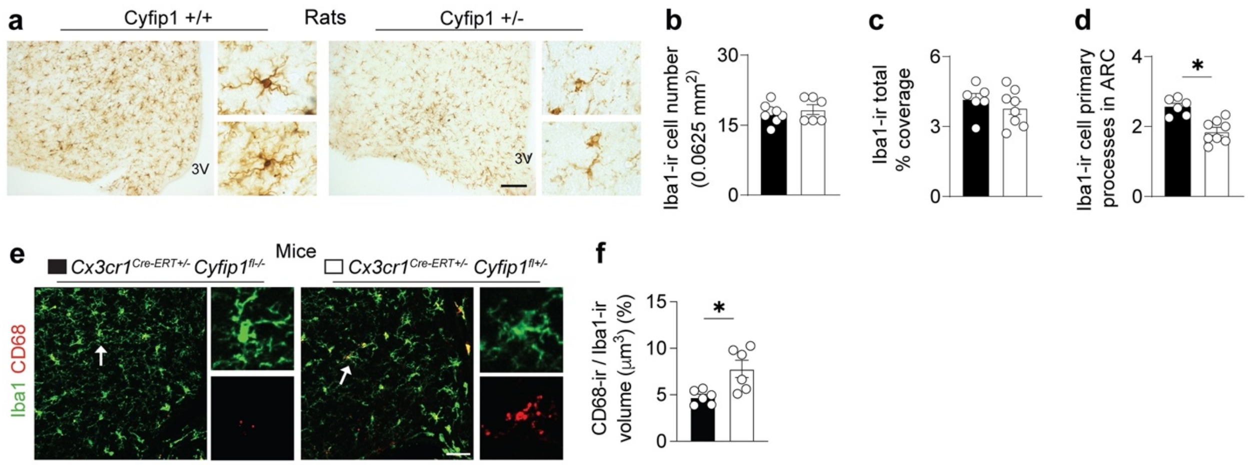

Supplementary Fig. 7

Microglial morphology in rats or mice with *Cyfip1* haploinsufficiency. a–d Immunohistochemistry for Iba1-ir microglia in the mediobasal hypothalamus of wild-type (n=7) or *Cyfip1* haploinsufficient (n=8) female rats. Dark arrow-pointed microglia in the left panel of each genotype in **a** are shown with higher magnification in the right panels. **e** Representative images of CD68 expression in Iba1-ir microglia in the mediobasal hypothalamus of control mice (*Cx3cr1*$^{Cre\text{-}ERT+/-}$ *Cyfip1*$^{fl-/-}$) (n=6) or *Cx3cr1*$^{Cre\text{-}ERT+/-}$ *Cyfip1*$^{fl+/-}$ mice (n=6). White arrow-pointed microglia in the left panel in each genotype are shown at higher magnification in the right panels. **f** Quantitative analysis of CD68-ir volume relative to the Iba1-ir volume. Scale bar: 100 µm in **a**, 50 µm in **e**. Data are represented as mean ± SEM. Significance in **d** was calculated using the Student's t-test. * $p < 0.05$.

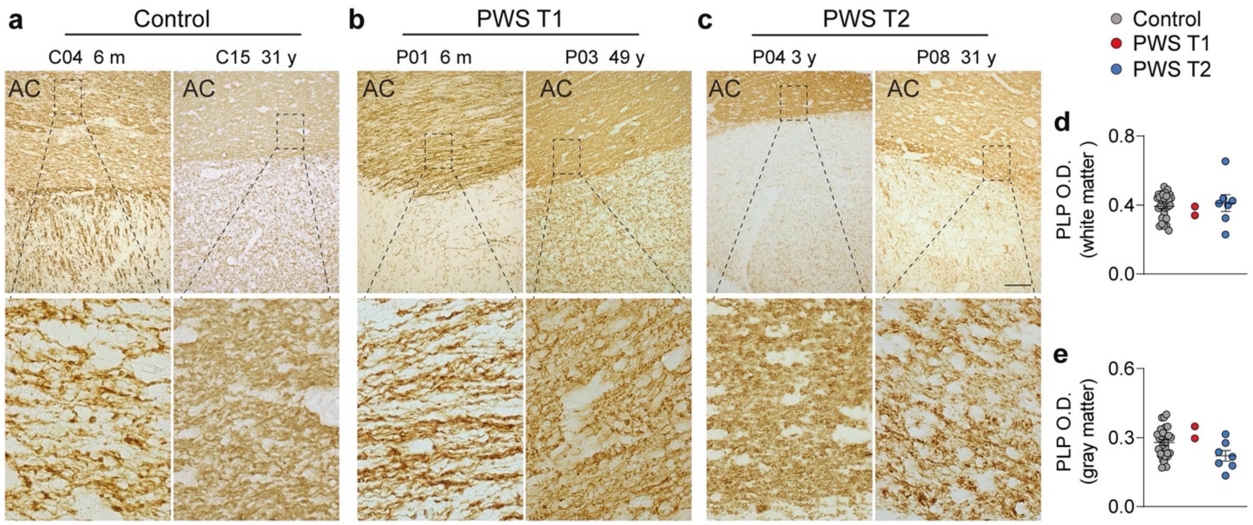

Supplementary Fig. 8

White matter microstructure of the anterior commissure is normal in PWS subjects. **a-c** Representative images of PLP-ir in the anterior commissure of the control (n=32), PWS T1 (n=3), and PWS T2 (n=7) subjects. Framed areas in upper panels are shown in higher magnification in lower panels. **d, e** Quantitative analysis of PLP-ir optical density in the white matter or gray matter. AC, anterior commissure; O.D., optical density; m, months; y, years. Scale bar: 100 µm in the **a-c** upper panel and 20 µm in the lower panel. Data are represented as mean ± SEM.

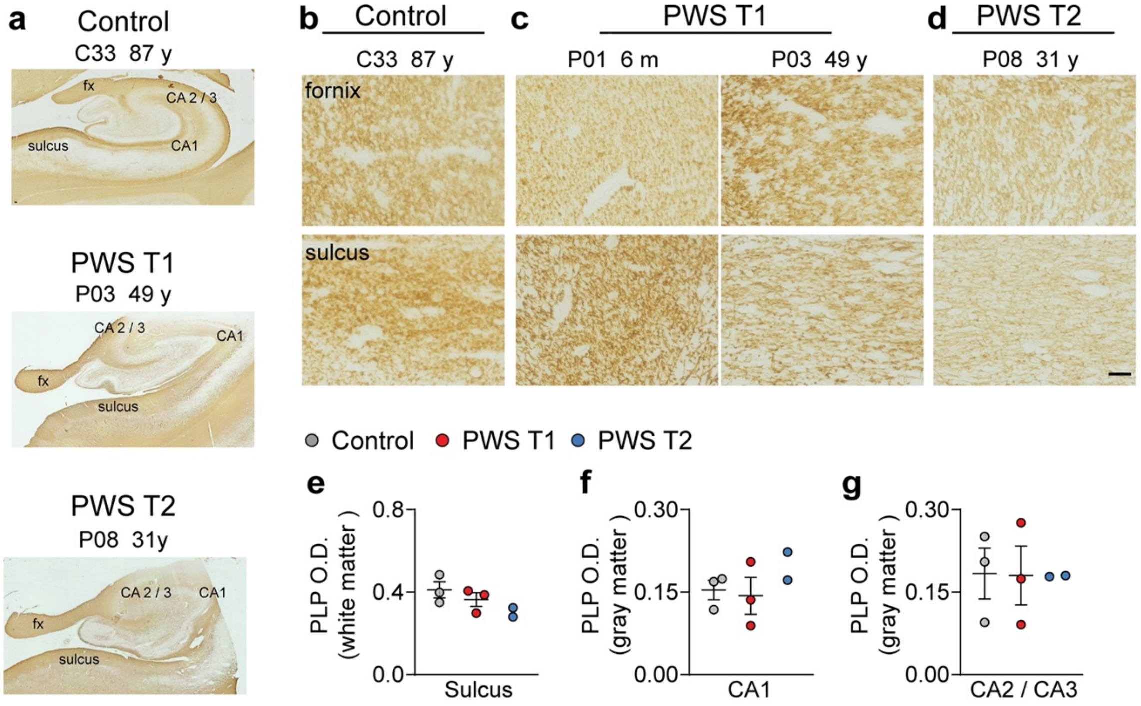

Supplementary Fig. 9

Intact white matter microstructure in PWS hippocampus. a Overview of PLP-ir in coronal sections of the hippocampus of control and PWS subjects. **b-d** Representative images of PLP-ir in the white matter of the hippocampus of control (n=3), PWS T1 (n=3) and PWS T2 (n=2) subjects. In contrast to PLP-ir in the hypothalamus, PLP-ir white matter in the hippocampus presents a normal microstructure. **e-g** Quantitative parameters of PLP-ir in the hippocampal sulcus, CA1 and CA2/CA3. fx, fornix; O.D., optical density; m, months; y, years. Scale bar: 20 µm in **b-d.** Data are represented as mean ± SEM.

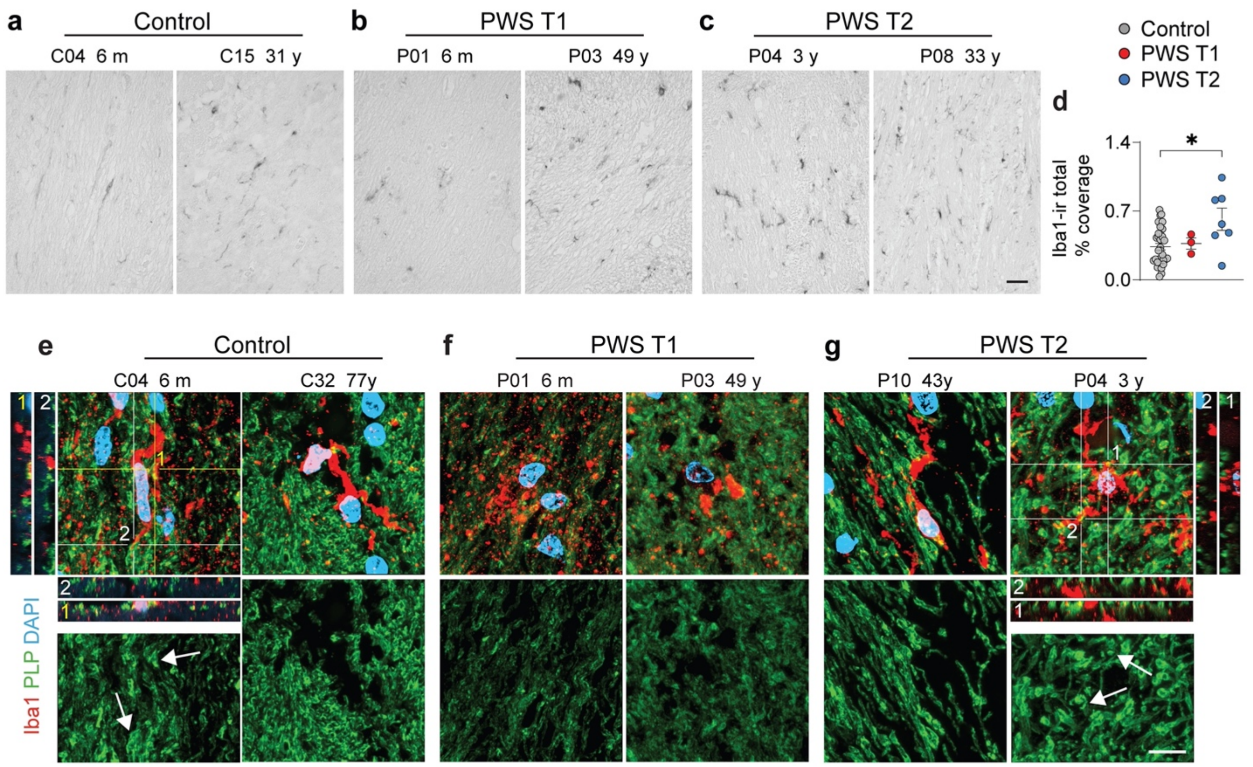

Supplementary Fig. 10

Increased microglial cells in the fornix of PWS T2 subjects and the few PLP taken up by microglia in the fornix. **a-c** Representative images of Iba1-ir cells within the fornix of control (n=32), PWS T1 (n=3), and PWS T2 (n=7) subjects. White matter Iba1-ir cells also showed morphological disruption in the PWS T1. **d** Quantitative analysis of Iba1-ir relative area of coverage in the fornix. **e-g** Illustrative images of co-staining of PLP-ir and Iba1-ir within the bounds of the fornix of control, PWS T1 and PWS T2 individuals. The lower panels show single PLP-ir. In subject C04 or P04, the presence of PLP-ir inside or near the Iba1-ir microglia was illustrated by two orthogonal views, respectively. In the lower panel of C04, white arrows point to spot 1 where the PLP-ir is inside the microglia and to spot 2 where the PLP-ir is near the microglia. Similarly, in the lower panel of P04, white arrows point to two spots where the PLP-ir is near the microglia. m, months; y, years. Scale bar: 20 µm in **a**-**c**; 10 µm in **e**-**g**. Data are represented as mean ± SEM. Significance was calculated using Kruskal–Wallis test in **d**. * $p < 0.05$.

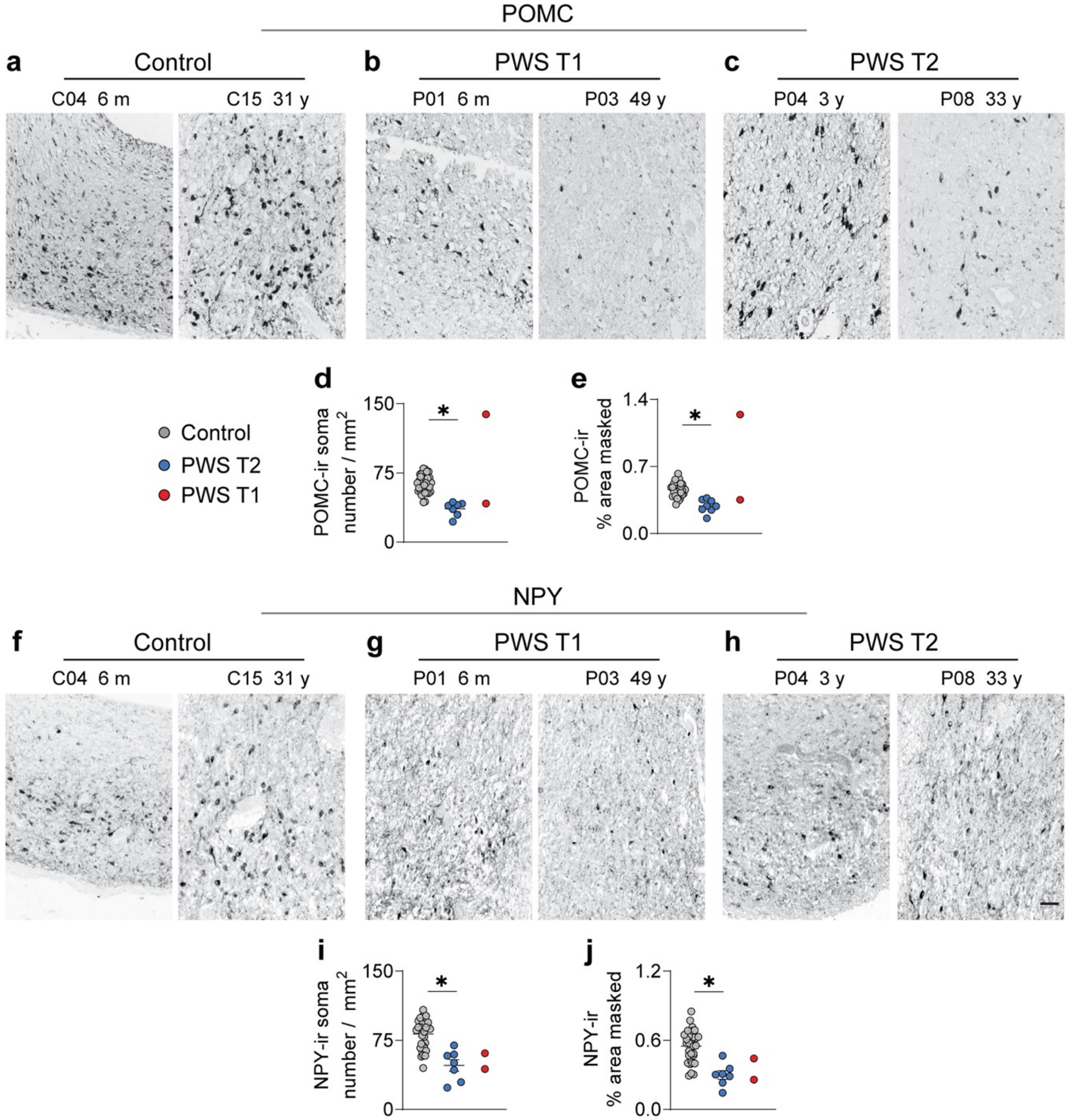

Supplementary Fig. 11

PWS is associated with a reduction in POMC-ir and NPY-ir neurons. a-c Representative images of POMC-ir neurons in the infundibular nucleus of control (n=32), PWS T1 (n=2) and PWS T2 (n=3) subjects. **d**, **e** Quantitative analysis of neuronal parameters indicating POMC-ir soma number/mm^2 and relative area of coverage. **f-h** Representative images of NPY-ir neurons in control (n=32), PWS T1 (n=2) and PWS T2 (n=7) subjects. **i**, **j** Parameters of NPY-ir cells regarding soma number / mm^2 and relative area of coverage. m, months; y, years. Scale bar: 50 µm in **a**-**c** and **h**-**j**. Data are represented as mean ± SEM. Significance was calculated using Kruskal–Wallis test in **d**, **e**, **i** and **j**. * $p < 0.05$.

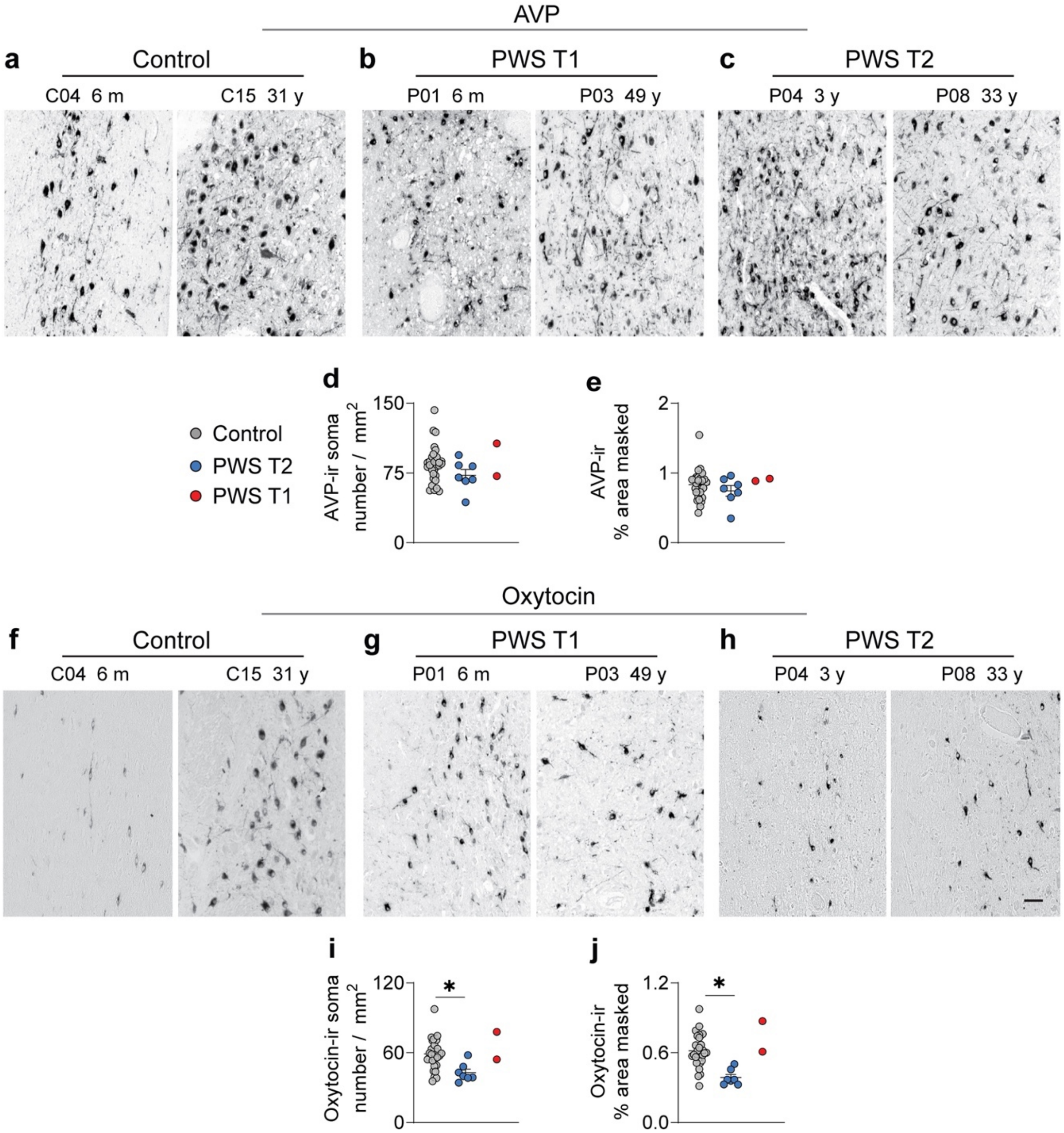

Supplementary Fig. 12

Reduction in oxytocin-ir neurons in the paraventricular nucleus of the hypothalamus in PWS subjects. a-c Representative images of AVP-ir neurons in the paraventricular nucleus of the hypothalamus of control (n=30), PWS T1 (n=2) and PWS T2 (n=3) subjects. **d, e** Quantitative parameters of AVP-ir cells indicating the AVP-ir soma number/mm^2 and relative area coverage. **f-h** Representative images of oxytocin-ir neurons in the paraventricular nucleus of the hypothalamus in control (n=30), PWS T1 (n=2) and PWS T2 (n=7) subjects. **i**, **j** Comparison between oxytocin-ir neuron parameters and oxytocin-ir soma number/mm^2 and the relative area of coverage. m, months; y, years. Scale bar: 50 µm in a-c and h-j. Data are represented as mean ± SEM. Significance was calculated using Kruskal–Wallis test in d, e, i and j. * $p < 0.05$.

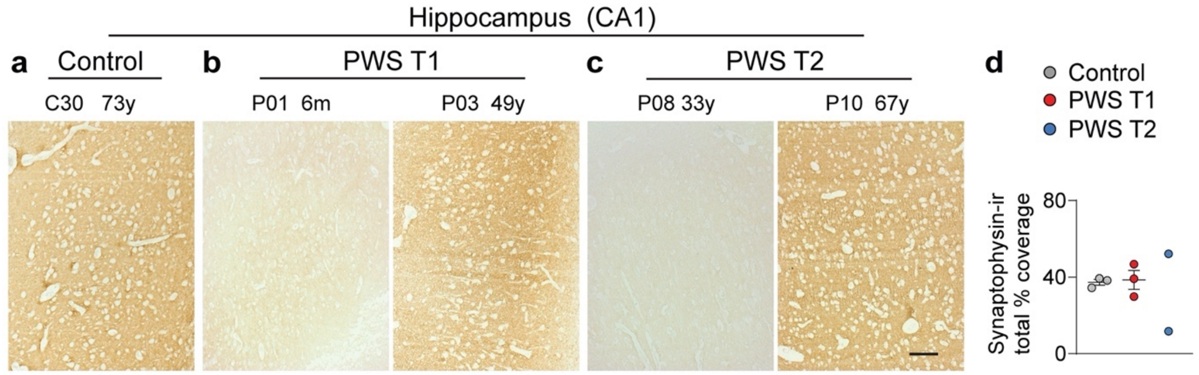

Supplementary Fig. 13

Hippocampal synaptophysin-ir is unaltered in PWS subjects. a-c Representative images of hippocampal (CA1) synaptophysin-ir in control (n=3), PWS T1 (n=3) and PWS T2 (n=2) subjects. **d** Quantification of CA1 synaptophysin-ir relative area of coverage. m, months; y, years. Scale bar: 50 µm in **a**-**c**. Data are represented as mean ± SEM.

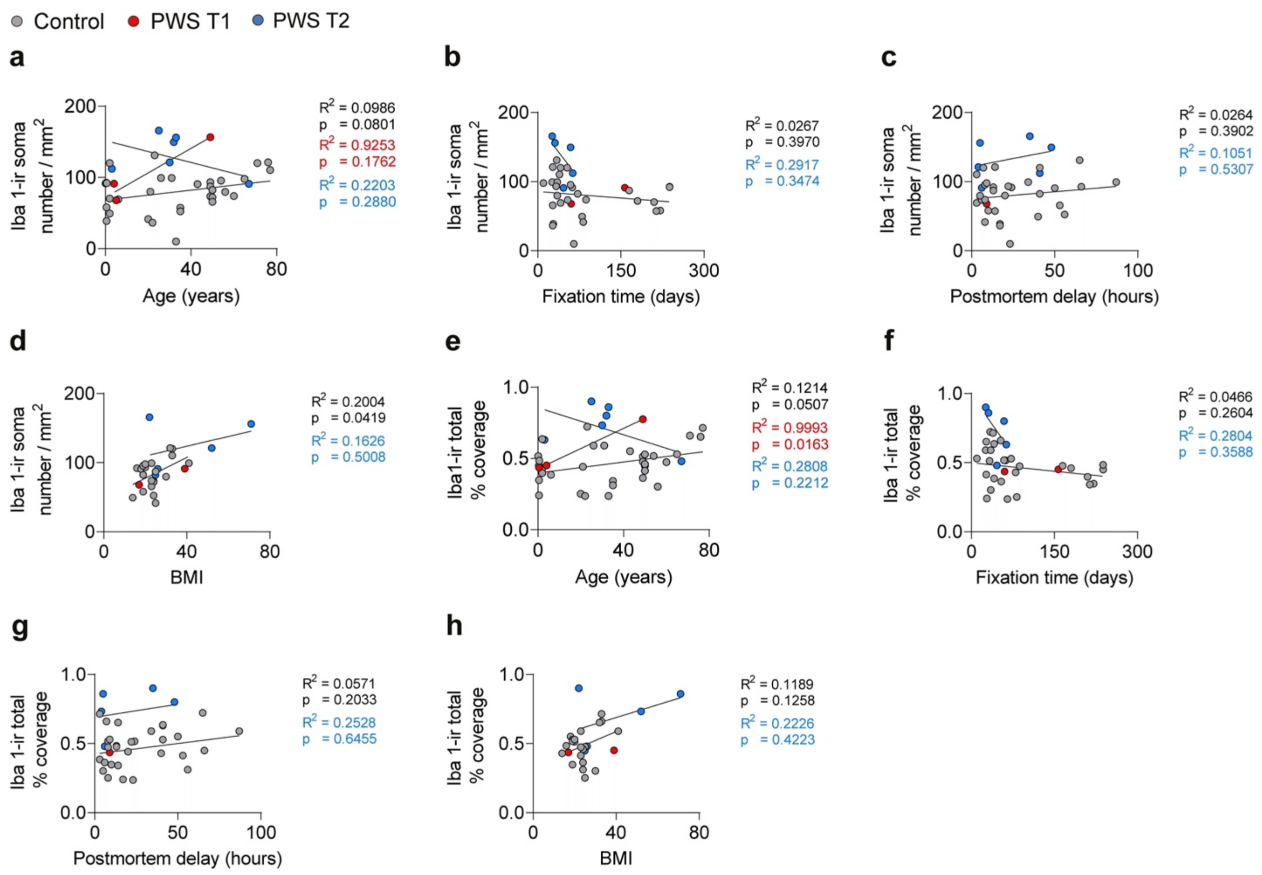

Supplementary Fig. 14

Confounder analysis of Iba1-ir in the hypothalamus of control and PWS subjects. **a-d** Plots of Iba1-ir soma number/mm^2 in the hypothalamus according to age, fixation time, postmortem delay and body mass index (BMI). **e-h** Plots of the Iba1-ir relative area of coverage in the mediobasal hypothalamus according to age, fixation time, postmortem delay and BMI.

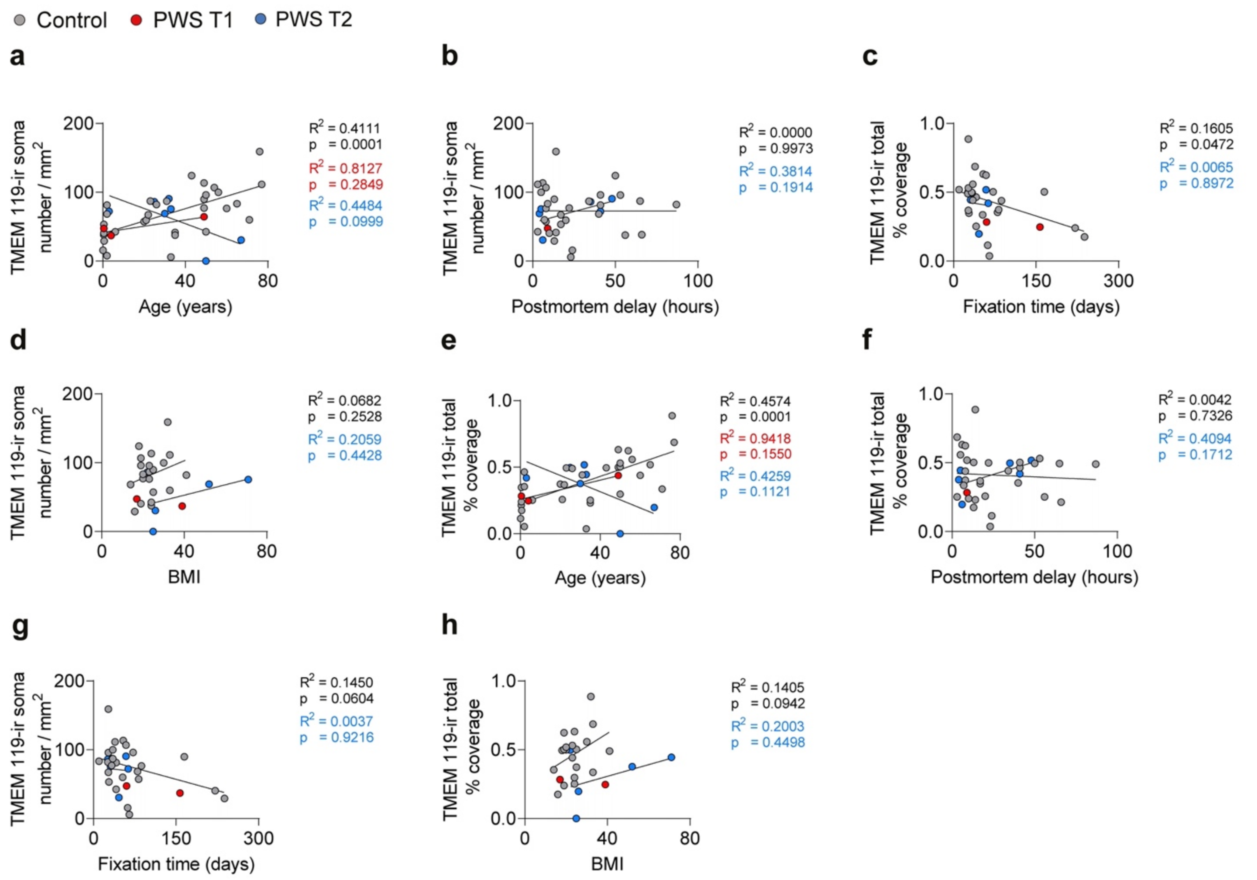

Supplementary Fig. 15

Confounder analysis of TMEM 119-ir in the hypothalamus of control and PWS subjects. a-d Plots of TMEM 119-ir soma number/mm^2 in the hypothalamus according to age, fixation time, postmortem delay and body mass index (BMI). **e-h** Plots of TMEM 119-ir relative area of coverage in the mediobasal hypothalamus according to age, fixation time, postmortem delay, and BMI.

.

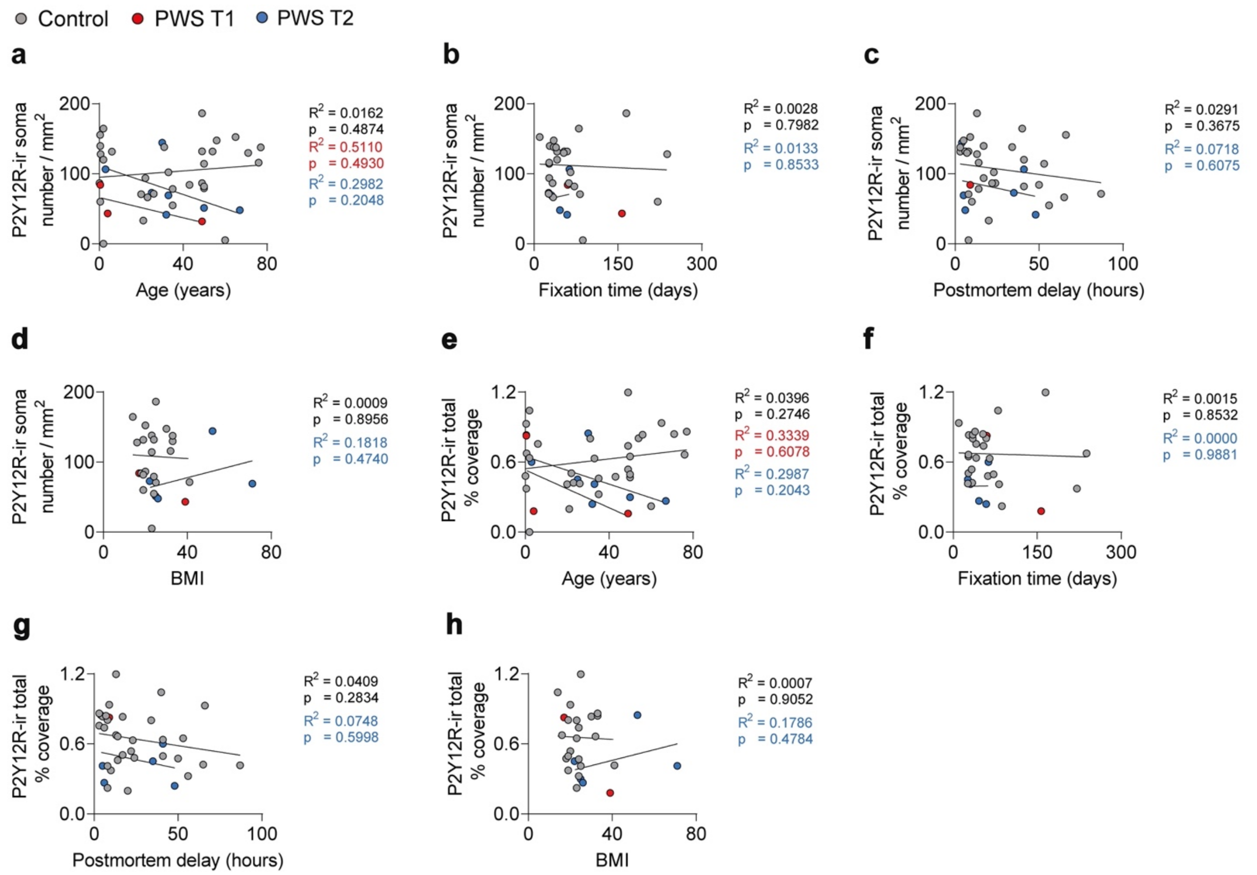

Supplementary Fig. 16

Confounder analysis of P2Y12R-ir in control and PWS subjects. a-d Plots of P2Y12R-ir soma number/mm^2 in the mediobasal hypothalamus according to age, fixation time, postmortem delay and body mass index (BMI). **e-h** Plots of P2Y12R-ir relative area of coverage in the mediobasal hypothalamus according to age, fixation time, postmortem delay and BMI.

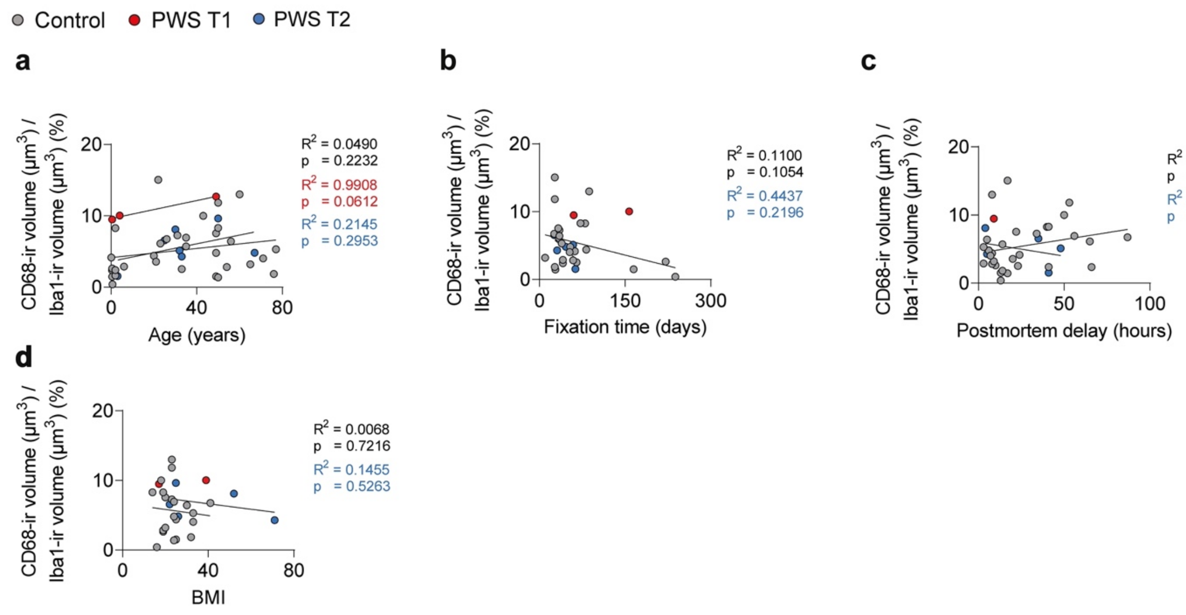

Supplementary Fig. 17

Confounder analysis of CD68-ir in control and PWS subjects. a-d Plots of volume percentage of CD68-ir in relation to Iba1-ir according to age, fixation time, postmortem delay and body mass index (BMI).

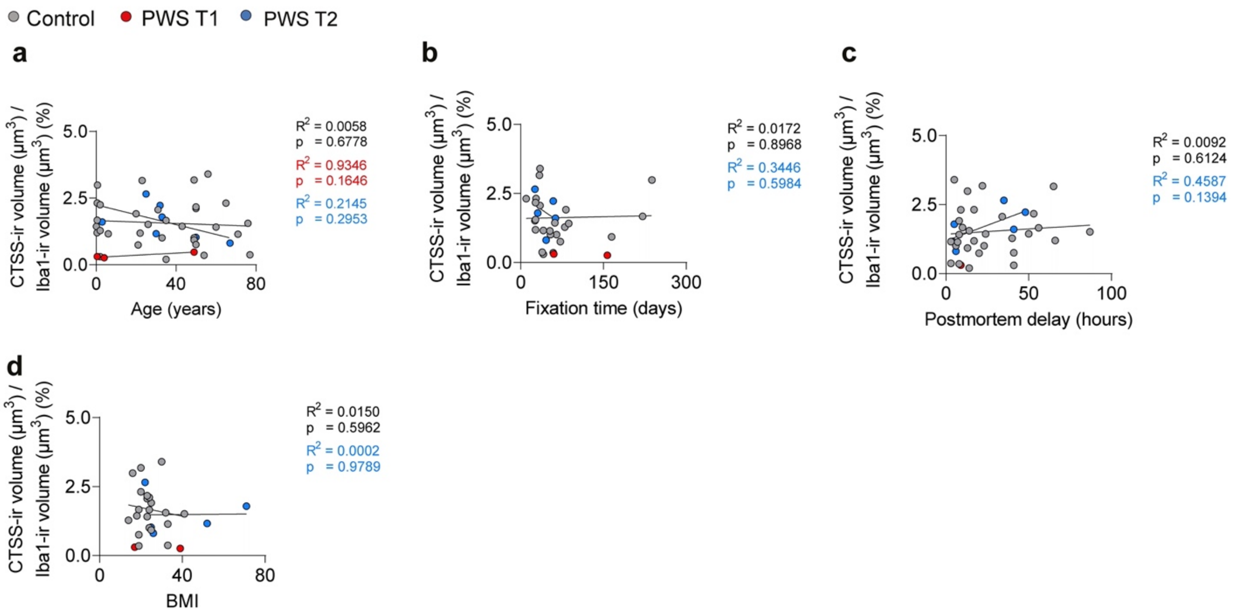

Supplementary Fig. 18

Confounder analysis of CTSS-ir in control and PWS subjects. a-d Plots of volume percentage of CTSS-ir in relation to Iba1-ir according to age, fixation time, postmortem delay and body mass index (BMI).

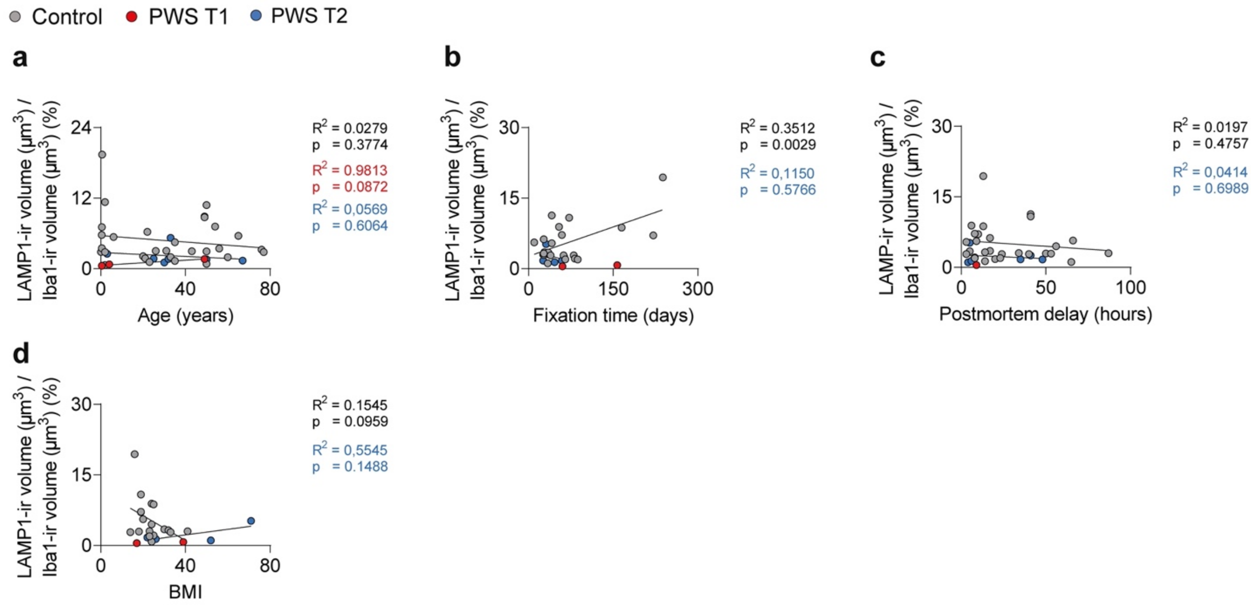

Supplementary Fig. 19

Confounder analysis of LAMP1-ir in control and PWS subjects. a-d Plots of volume percentage of LAMP1-ir in relation to Iba1-ir according to age, fixation time, postmortem delay and body mass index (BMI).

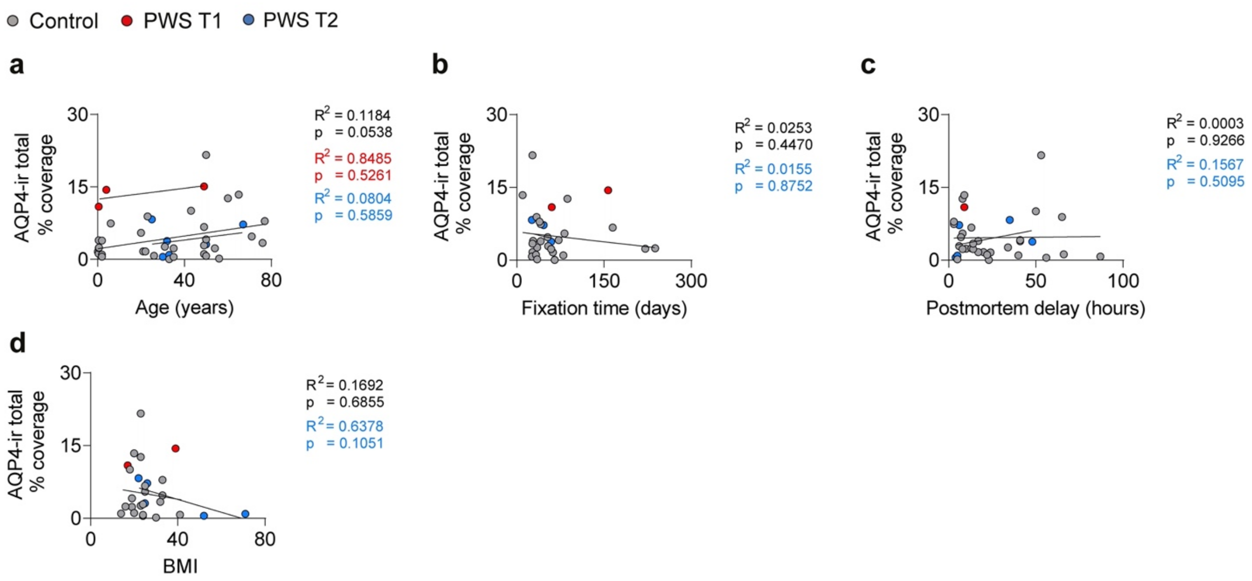

Supplementary Fig. 20

Confounder analysis of AQP4-ir in the hypothalamus of control and PWS subjects. a-d Plots of AQP4 relative area coverage according to age, fixation time, postmortem delay and body mass index (BMI).

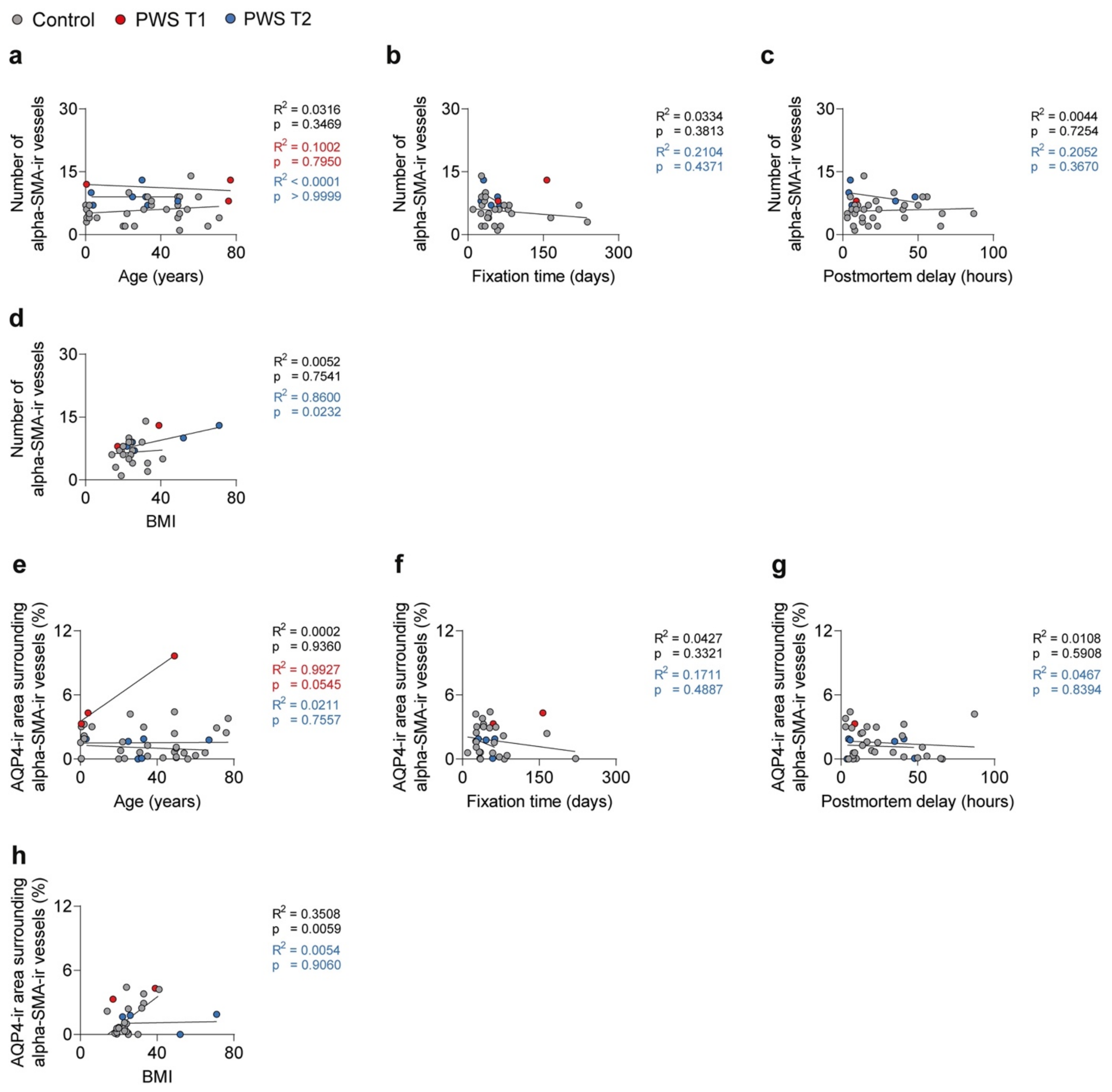

Supplementary Fig. 21

Confounder analysis of alpha-SMA-ir and the AQP4-ir in the perivascular space in the hypothalamus of control and PWS subjects. a-d Plots of alpha-SMA relative area coverage according to age, fixation time, postmortem delay and body mass index (BMI). **e-h** Plots of % of AQP4-ir surrounding the alpha-SMA vessels to age, fixation time, postmortem delay and BMI.

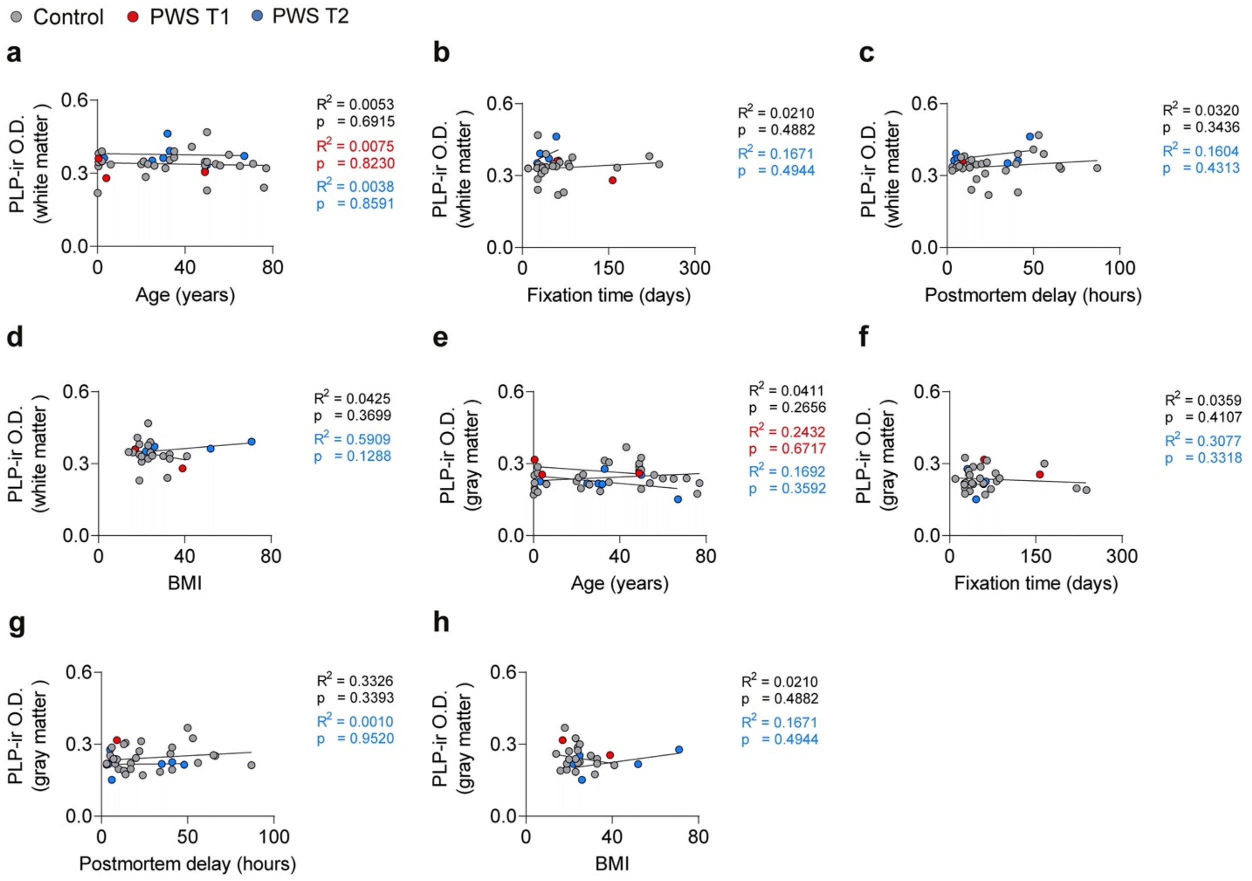

Supplementary Fig. 22

Confounder analysis of PLP-ir in the fornix of control and PWS subjects. a-d Plots of PLP-ir optical density within the white matter in the fornix according to age, fixation time, postmortem delay, and body mass index (BMI). **e-h** Plots of PLP-ir optical density within the gray matter adjacent to the fornix according to age, fixation time, postmortem delay and BMI.

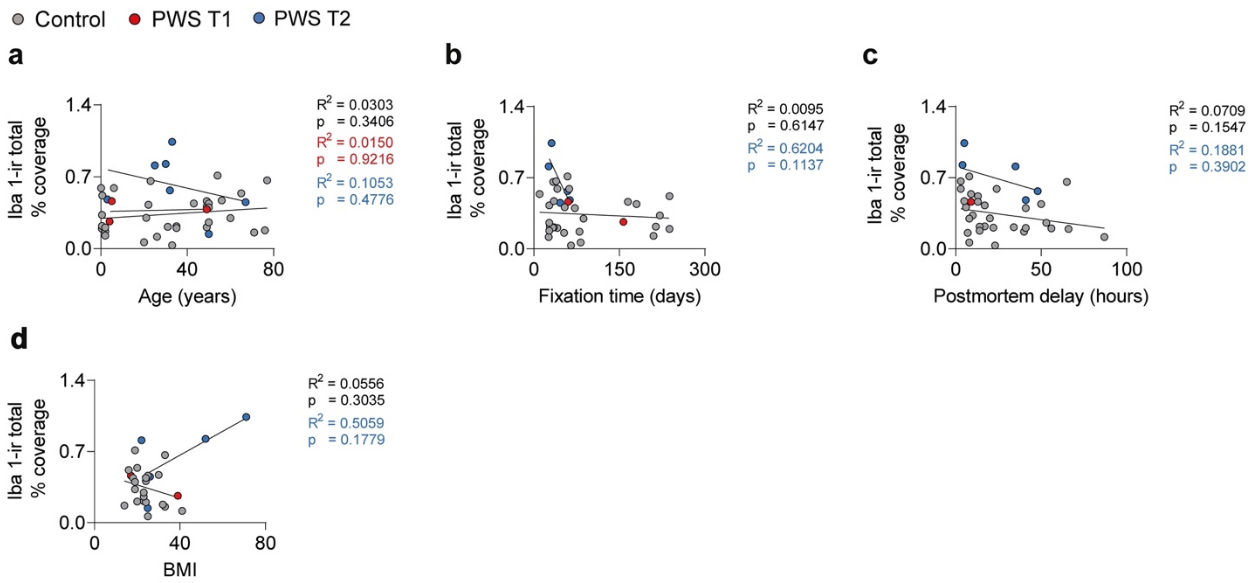

Supplementary Fig. 23

Confounder analysis of Iba1-ir in the fornix of control and PWS subjects. **a-d** Plots of Iba1-ir relative area of coverage in the fornix according to age, fixation time, postmortem delay and body mass index (BMI).

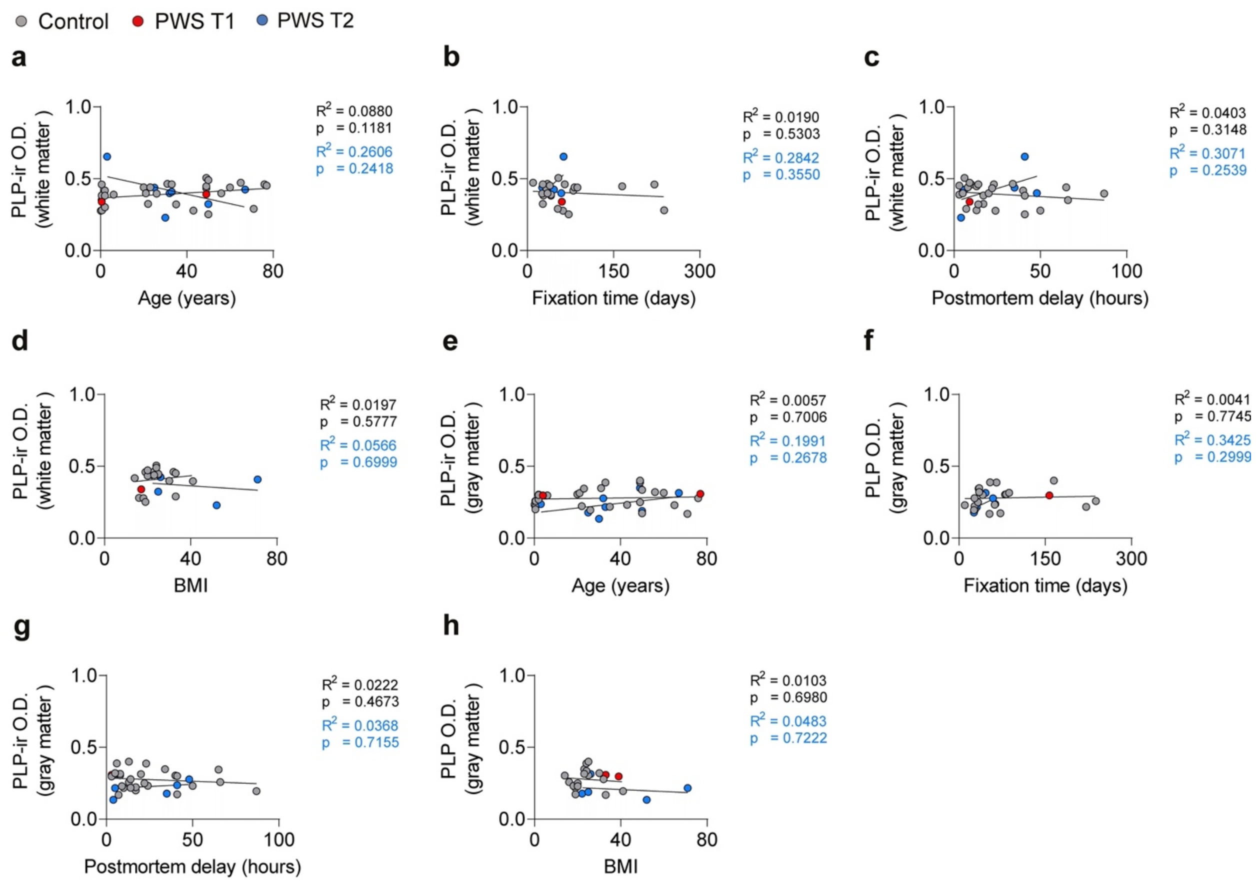

Supplementary Fig. 24

Confounder analysis of PLP-ir in the anterior commissure in control and PWS subjects. a-d Plots of PLP-ir optical density within the white matter in the anterior commissure according to age, fixation time, postmortem delay, and body mass index (BMI). **e-h** Plots of PLP-ir optical density within the gray matter adjacent to the anterior commissure according to age, fixation time, postmortem delay and BMI.

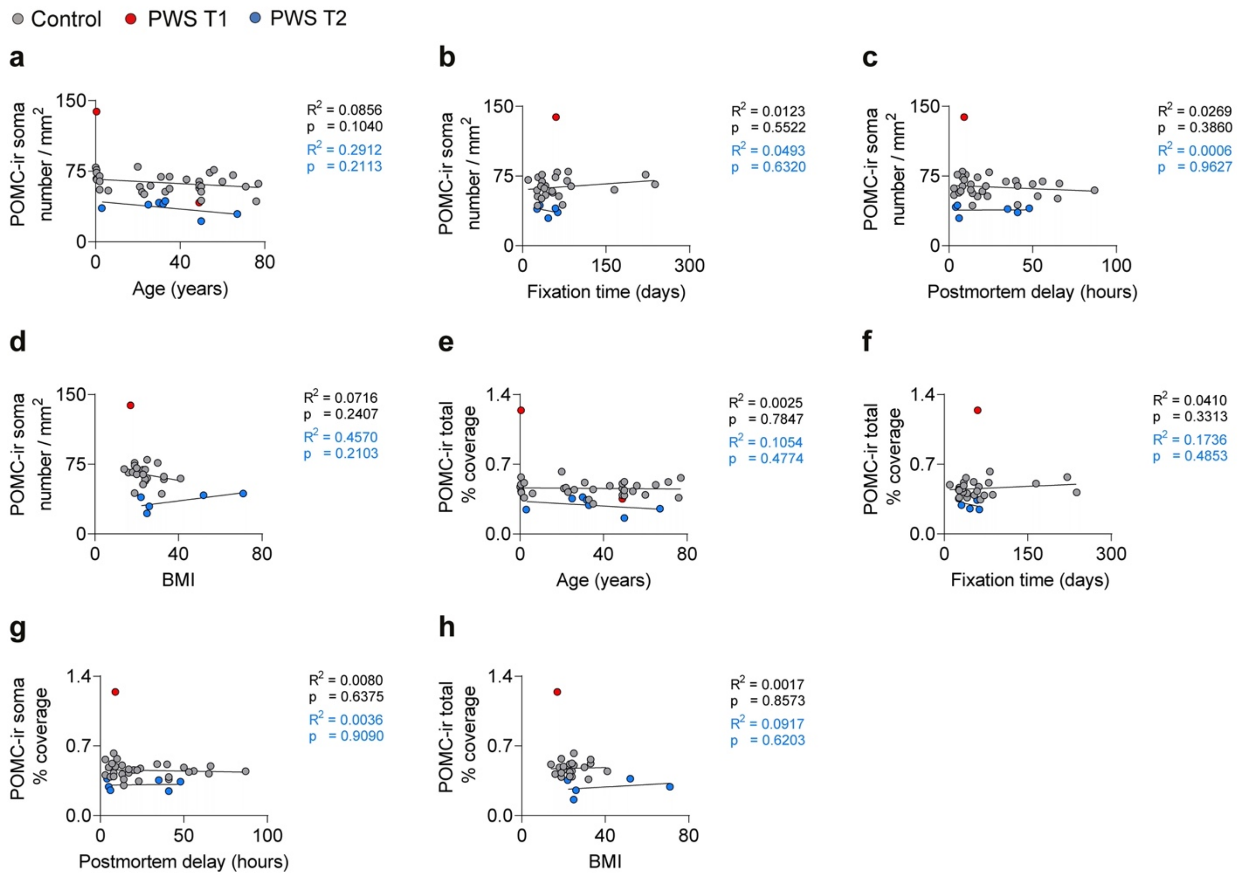

Supplementary Fig. 25

Confounder analysis of POMC-ir neurons in the infundibular nucleus of control and PWS subjects. a-d Plots of POMC-ir soma number/mm^2 according to age, fixation time, postmortem delay and body mass index (BMI). **e-h** Plots of POMC-ir relative area of coverage according to age, fixation time, postmortem delay and BMI.

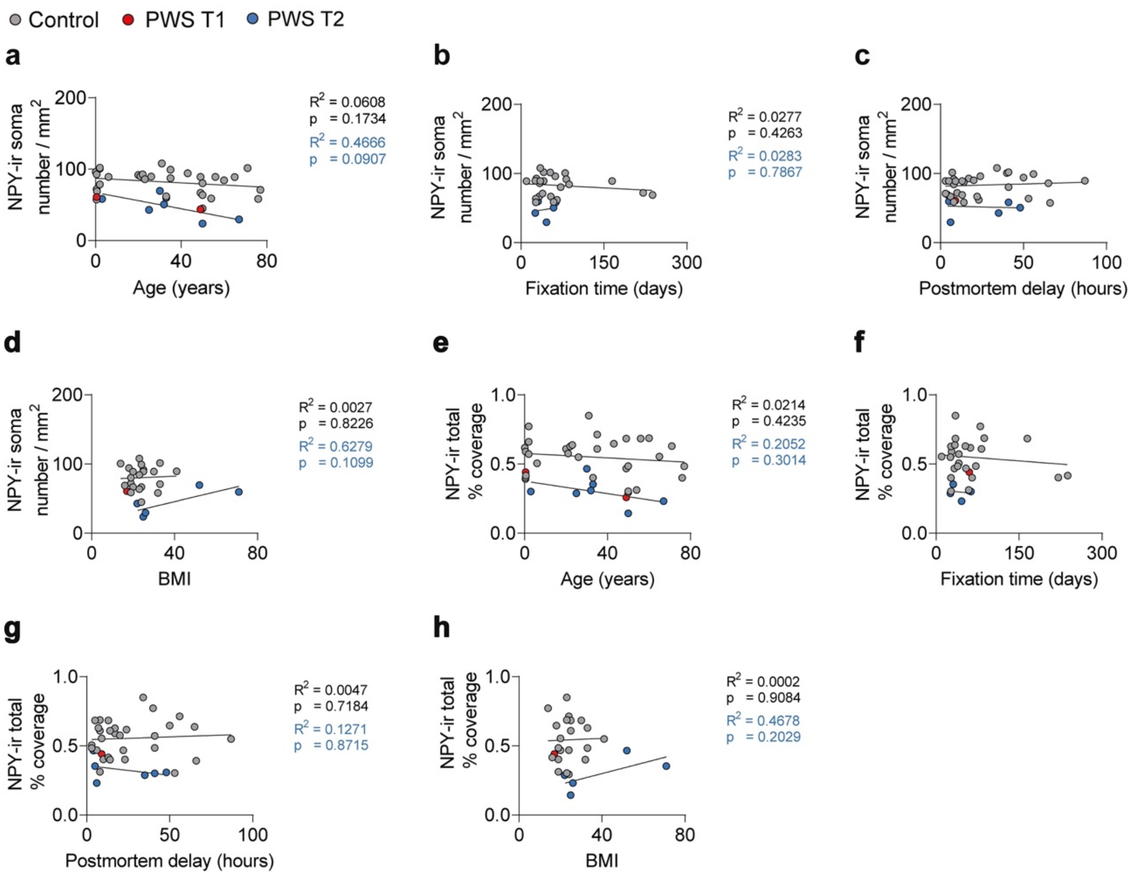

Supplementary Fig. 26

Confounder analysis of NPY-ir neurons in the infundibular nucleus of control and PWS subjects. a-d Plots of NPY-ir soma number/mm^2 according to age, fixation time, postmortem delay and body mass index (BMI). **e-h** Plots of NPY-ir relative area of coverage according to age, fixation time, postmortem delay and BMI.

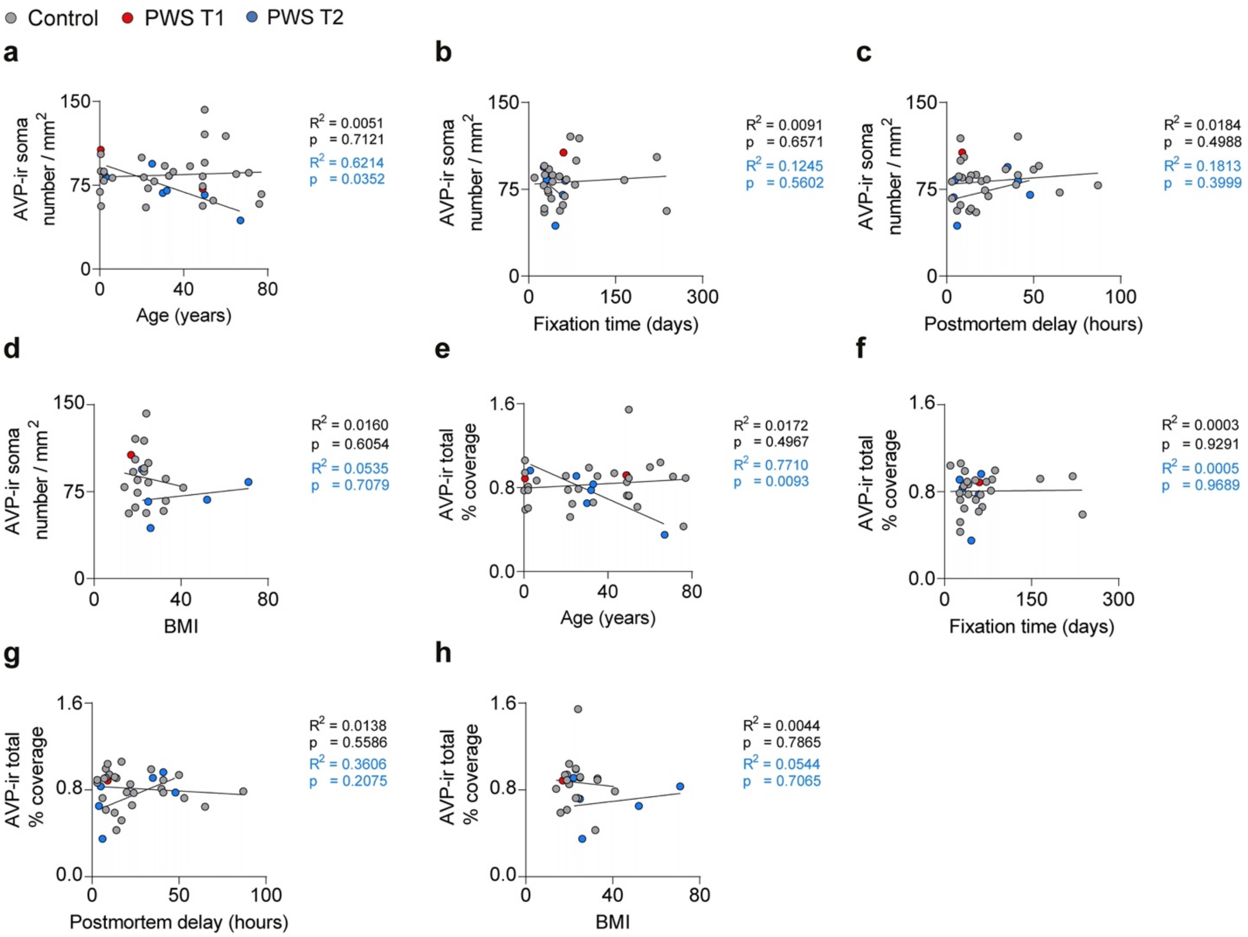

Supplementary Fig. 27

Confounder analysis of AVP-ir neurons in the paraventricular nucleus of the hypothalamus in control and PWS subjects. a-d Plots of AVP-ir soma number/mm^2 according to age, fixation time, postmortem delay, and body mass index (BMI). **e-h** Plots of AVP-ir relative area of coverage according to age, fixation time, postmortem delay and BMI.

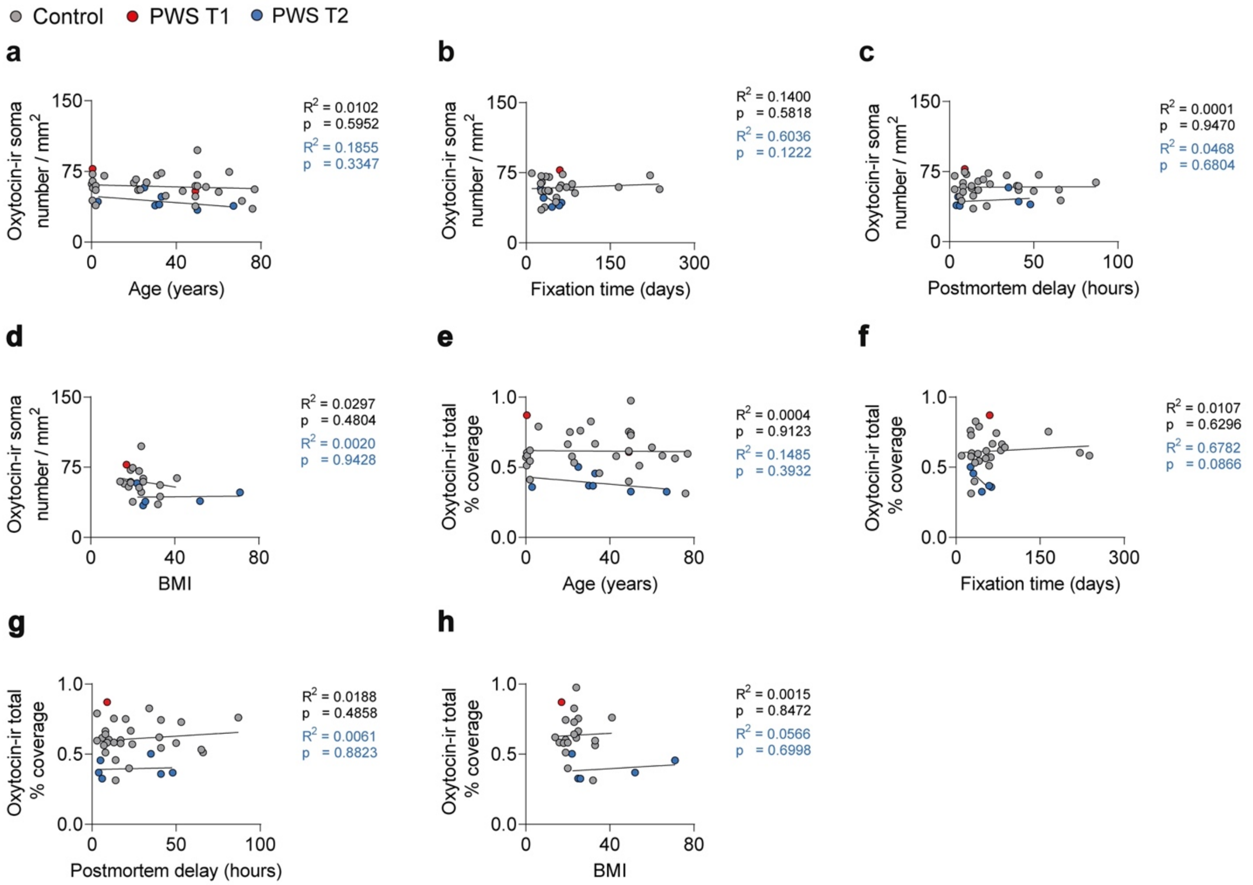

Supplementary Fig. 28

Confounder analysis of oxytocin-ir neurons in the paraventricular nucleus of the hypothalamus in control and PWS subjects. a-d Plots of oxytocin-ir soma number/mm^2 according to age, fixation time, postmortem delay, and body mass index (BMI). **e-h** Plots of the oxytocin-ir relative area of coverage according to age, fixation time, postmortem delay and BMI.

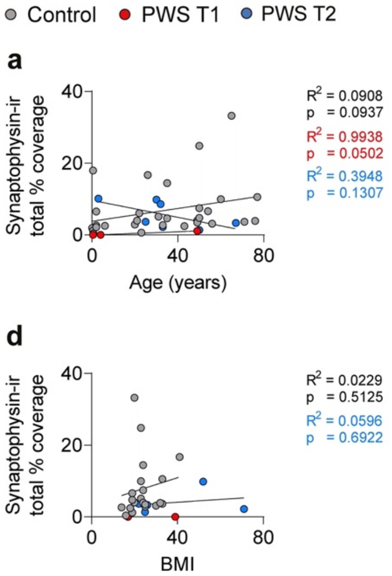

Supplementary Fig. 29

Confounder analysis of synaptophysin-ir in the hypothalamus of control and PWS subjects. a-d Plots of synaptophysin-ir relative area of coverage according to age, fixation time, postmortem delay and BMI.
